# Author Correction: The challenge of mapping the human connectome based on diffusion tractography

**DOI:** 10.1038/s41467-019-12867-2

**Published:** 2019-11-04

**Authors:** Klaus H. Maier-Hein, Peter F. Neher, Jean-Christophe Houde, Marc-Alexandre Côté, Eleftherios Garyfallidis, Jidan Zhong, Maxime Chamberland, Fang-Cheng Yeh, Ying-Chia Lin, Qing Ji, Wilburn E. Reddick, John O. Glass, David Qixiang Chen, Yuanjing Feng, Chengfeng Gao, Ye Wu, Jieyan Ma, Renjie He, Qiang Li, Carl-Fredrik Westin, Samuel Deslauriers-Gauthier, J. Omar Ocegueda González, Michael Paquette, Samuel St-Jean, Gabriel Girard, François Rheault, Jasmeen Sidhu, Chantal M. W. Tax, Fenghua Guo, Hamed Y. Mesri, Szabolcs Dávid, Martijn Froeling, Anneriet M. Heemskerk, Alexander Leemans, Arnaud Boré, Basile Pinsard, Christophe Bedetti, Matthieu Desrosiers, Simona Brambati, Julien Doyon, Alessia Sarica, Roberta Vasta, Antonio Cerasa, Aldo Quattrone, Jason Yeatman, Ali R. Khan, Wes Hodges, Simon Alexander, David Romascano, Muhamed Barakovic, Anna Auría, Oscar Esteban, Alia Lemkaddem, Jean-Philippe Thiran, H. Ertan Cetingul, Benjamin L. Odry, Boris Mailhe, Mariappan S. Nadar, Fabrizio Pizzagalli, Gautam Prasad, Julio E. Villalon-Reina, Justin Galvis, Paul M. Thompson, Francisco De Santiago Requejo, Pedro Luque Laguna, Luis Miguel Lacerda, Rachel Barrett, Flavio Dell’Acqua, Marco Catani, Laurent Petit, Emmanuel Caruyer, Alessandro Daducci, Tim B. Dyrby, Tim Holland-Letz, Claus C. Hilgetag, Bram Stieltjes, Maxime Descoteaux

**Affiliations:** 1 Division of Medical Image Computing, German Cancer Research Center (DKFZ), Heidelberg, 69120 Germany; 2 Sherbrooke Connectivity Imaging Lab (SCIL), Université de Sherbrooke, Sherbrooke, QC J1K 0A5 QC Canada; 3 Department of Intelligent Systems Engineering, School of Informatics and Computing, Indiana University, Bloomington, IN 47408 USA; 4 Krembil Research Institute, University Health Network, Toronto, Canada M5G 2C4; 5 Department of Neurological Surgery, University of Pittsburgh School of Medicine, Pittsburgh, PA 15213 USA; 6 IMT—Institute for Advanced Studies, Lucca, 55100 Italy; 7 Department of Diagnostic Imaging, St. Jude Children’s Research Hospital, Memphis, TN 38105 USA; 8 University of Toronto Institute of Medical Science, Toronto, Canada M5S 1A8; 9 Institute of Information Processing and Automation, Zhejiang University of Technology, Hangzhou, 310023 Zhejiang, China; 10 United Imaging Healthcare Co, Shanghai, 201807 China; 11 Shanghai Advanced Research Institute, Shanghai, 201210 China; 12 Laboratory of Mathematics in Imaging, Harvard Medical School, Boston, MA 02215 USA; 13 Center for Research in Mathematics, Guanajuato, 36023 Mexico; 14 PROVIDI Lab, Image Sciences Institute, University Medical Center Utrecht, Utrecht, 3508 The Netherlands; 15 Cardiff University Brain Research Imaging Centre, School of Psychology, Cardiff University, Maindy Road, Cardiff, CF24 4HQ UK; 16 Department of Radiology, University Medical Center Utrecht, Utrecht, 3508 The Netherlands; 17 Centre de recherche institut universitaire de geriatrie de Montreal (CRIUGM), Université de Montréal, Montreal, QC Canada H3W 1W5; 18 Sorbonne Universités, UPMC Univ Paris 06, CNRS, INSERM, Laboratoire d’Imagerie Biomédicale (LIB), 75013 Paris, France; 19 Center for Advanced Research in Sleep Medicine, Hôpital du Sacré-Coeur de Montréal, Montreal, Canada H4J 1C5; 20 Neuroimaging Unit, Institute of Bioimaging and Molecular Physiology (IBFM), National Research Council (CNR), Policlinico Magna Graecia, Germaneto, 88100 CZ Italy; 21 Institute of Neurology, University Magna Graecia, Germaneto, 88100 CZ Italy; 22 Institute for Learning & Brain Sciences and Department of Speech & Hearing Sciences, University of Washington, Seattle, WA 98195 USA; 23 Departments of Medical Biophysics & Medical Imaging, Schulich School of Medicine and Dentistry, Western University, 1151 Richmond St N, London, ON Canada N6A 5C1; 24 Synaptive Medical Inc., MaRS Discovery District, 101 College Street, Suite 200, Toronto, ON Canada M5V 3B1; 25 Signal Processing Lab (LTS5), Ecole Polytechnique Federale de Lausanne, Lausanne, 1015 Switzerland; 26 Biomedical Image Technologies (BIT), ETSI Telecom., U. Politécnica de Madrid and CIBER-BBN, Madrid, 28040 Spain; 27 Department of Radiology, University Hospital Center (CHUV) and University of Lausanne (UNIL), Lausanne, 1011 Switzerland; 28 Medical Imaging Technologies, Siemens Healthcare, Princeton, NJ 08540 USA; 29 Imaging Genetics Center, Stevens Neuro Imaging and Informatics Institute, Keck School of Medicine of USC, Marina del Rey, CA 90033 USA; 30 NatBrainLab, Institute of Psychiatry, Psychology & Neuroscience, King’s College London, London, SE5 8AF UK; 31 Groupe d’imagerie Neurofonctionnelle—Institut des Maladies Neurodégénératives (GIN-IMN), UMR5293 CNRS, CEA, University of Bordeaux, Bordeaux, 33000 France; 32 Centre national de la recherche scientifique (CNRS), Institute for Research in IT and Random Systems (IRISA), UMR 6074 VISAGES Project-Team, Rennes, 35042 France; 33 Danish Research Centre for Magnetic Resonance, Center for Functional and Diagnostic Imaging and Research, Copenhagen University Hospital Hvidovre, Hvidovre, 2650 Denmark; 34 Department of Applied Mathematics and Computer Science, Technical University of Denmark, Kongens Lyngby, 2800 Denmark; 35 Division of Biostatistics, German Cancer Research Center (DKFZ), Heidelberg, 69120 Germany; 36 Department of Computational Neuroscience, University Medical Center Eppendorf, Hamburg, 20246 Germany; 37 University Hospital Basel, Radiology & Nuclear Medicine Clinic, Basel, 4031 Switzerland

**Keywords:** Computational biology and bioinformatics, Neuroscience, Nervous system, Medical research

Correction to: *Nature Communications* 10.1038/s41467-017-01285-x, published online 7 November 2017.

The original version of this Article contained an error in the spelling of the author Renjie He, which was incorrectly given as H. Renjie. This has now been corrected in both the PDF and HTML versions of the Article.

