# Supplementary material for: The challenge of mapping the human connectome based on diffusion tractography
Source: Nat Commun. 2017 Nov 7;8:1349. doi: 10.1038/s41467-017-01285-x (PMC5677006; doi:10.1038/s41467-017-01285-x)
Supplement: Supplementary file 1 — Supplementary Information [file 41467_2017_1285_MOESM1_ESM.pdf]

Supplementary Figures

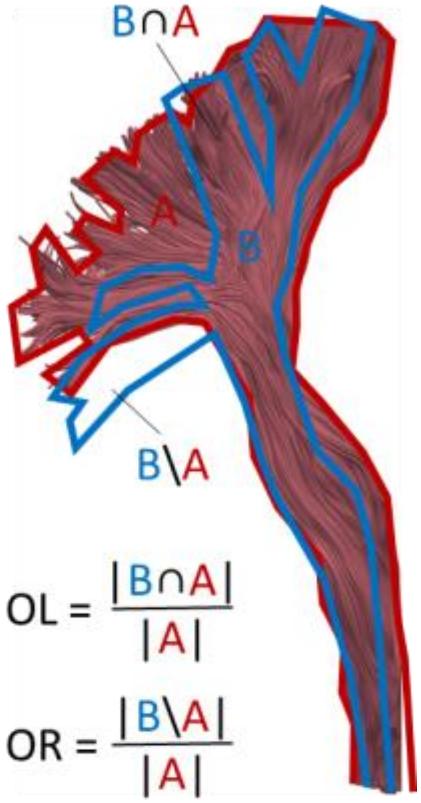

Supplementary Figure 1. Schematic illustration of the overlap (OL) and overreach (OR) metrics. OL quantifies the percent overlap of the ground truth bundle A (red) with the evaluated bundle B (blue). OR sets the non-overlapping volume of B into relation with the volume of the ground truth bundle A.

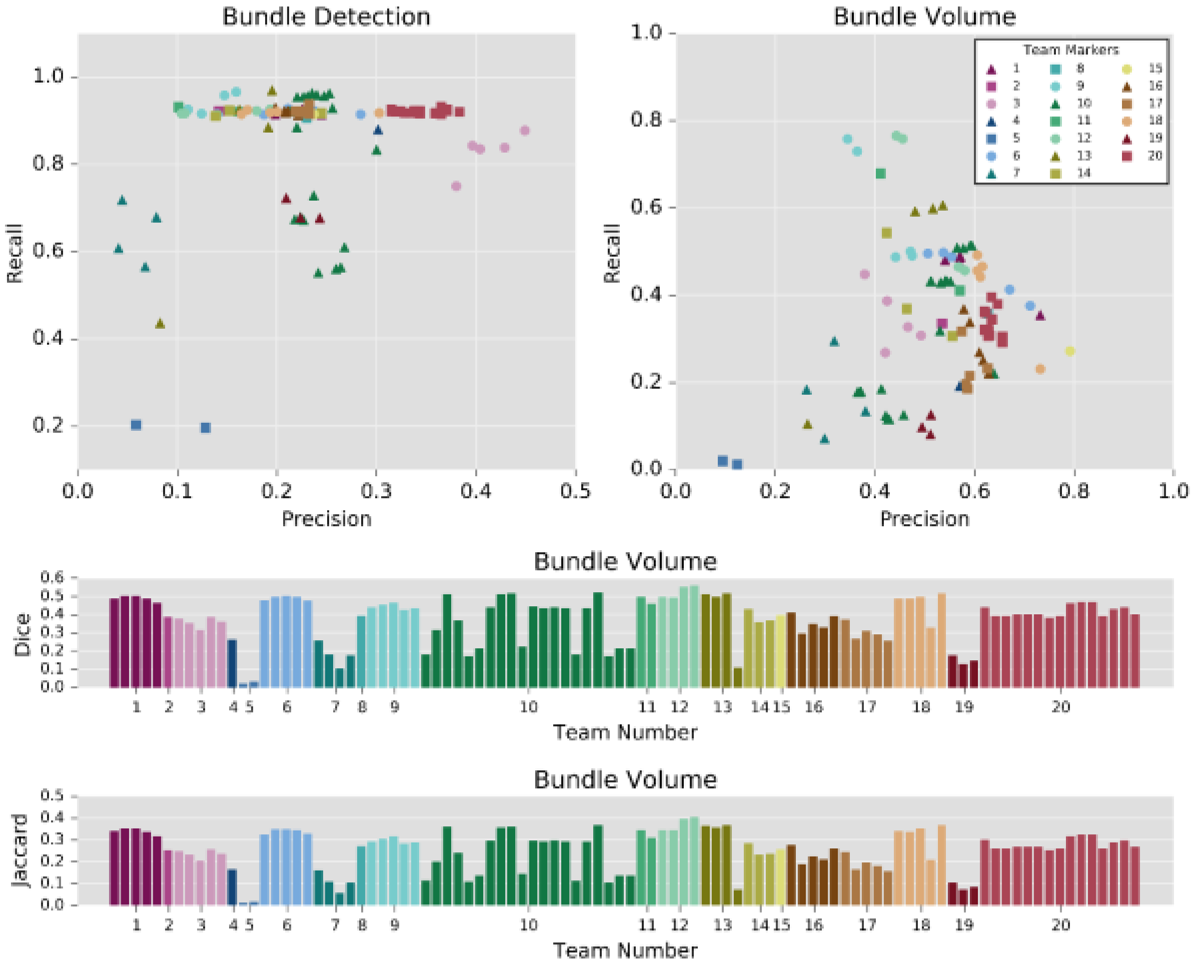

Supplementary Figure 2. Alternative metrics. This figure shows alternative metrics to the VB/IB and the OL/OR scores of Fig. 3 in the manuscript. Bundle detection rates are expressed in terms of precision and recall. To describe the bundle overlap, we also calculated the Dice coefficient and Jaccard index in addition to precision and recall.

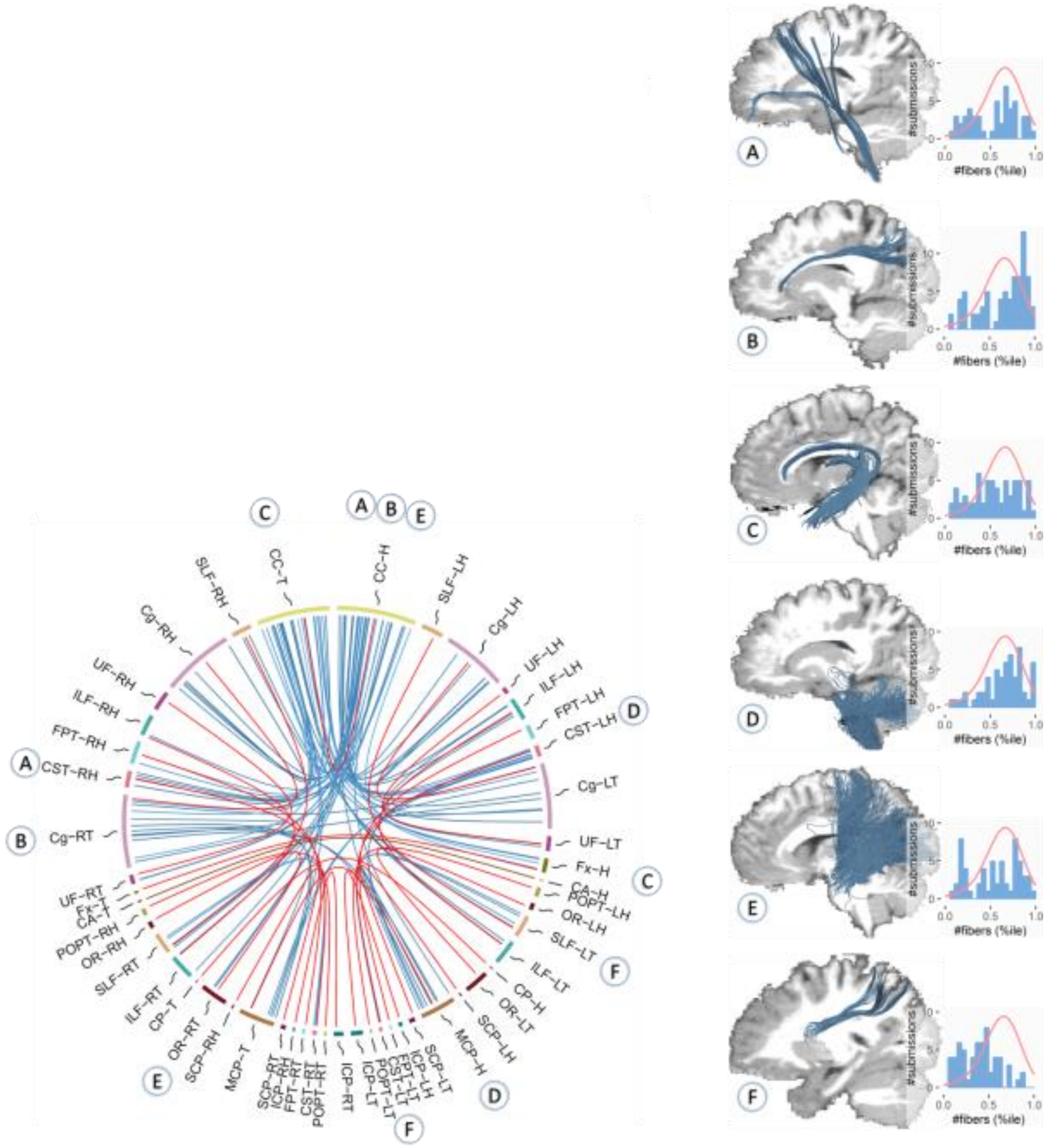

Supplementary Figure 3. Overview of valid (red) and invalid (blue) bundles. Each red or blue line in the connectivity wheel represents a valid (red) or invalid bundle (blue) detected by more than half of the submissions. The size and location of the segments has been chosen as in Supplementary Figure 1. Exemplary submissions that correspond to selected invalid bundles (A-F) are representatively shown in order to provide a notion of how coherent and “real” invalid bundles can appear. The blue histogram indicates the percentile fiber count that the bundle has received in the different submissions. The red line indicates the average distribution of percentile fiber counts in valid bundles, showing that the fiber counts of invalid bundles are similar to those of valid bundles.

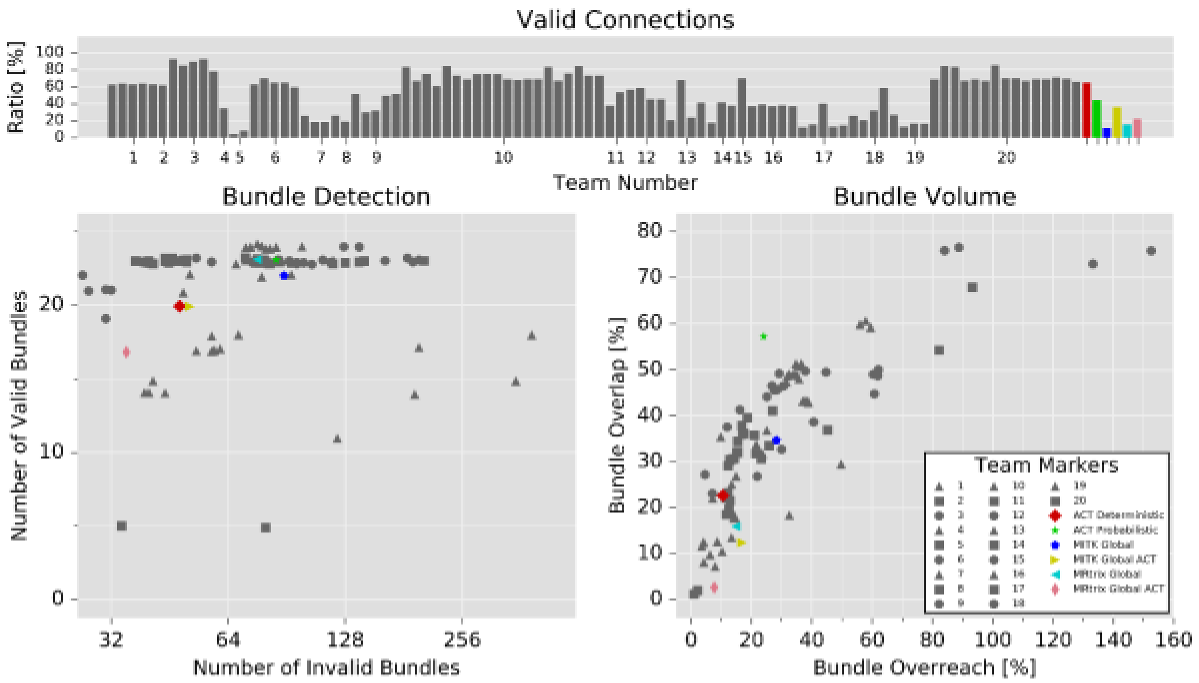

Supplementary Figure 4. Anatomically constrained and global tractography. This figure shows the scores of the original submissions (gray) in conjunction with the results of new experiments with global and anatomically constrained tractography (ACT, cf. Supplementary Note 2). Two toolkits were used to perform global tractography (MITK and MRtrix). Tissue segmentations were obtained from the simulated T1 weighted image using MRtrix (5ttgen).

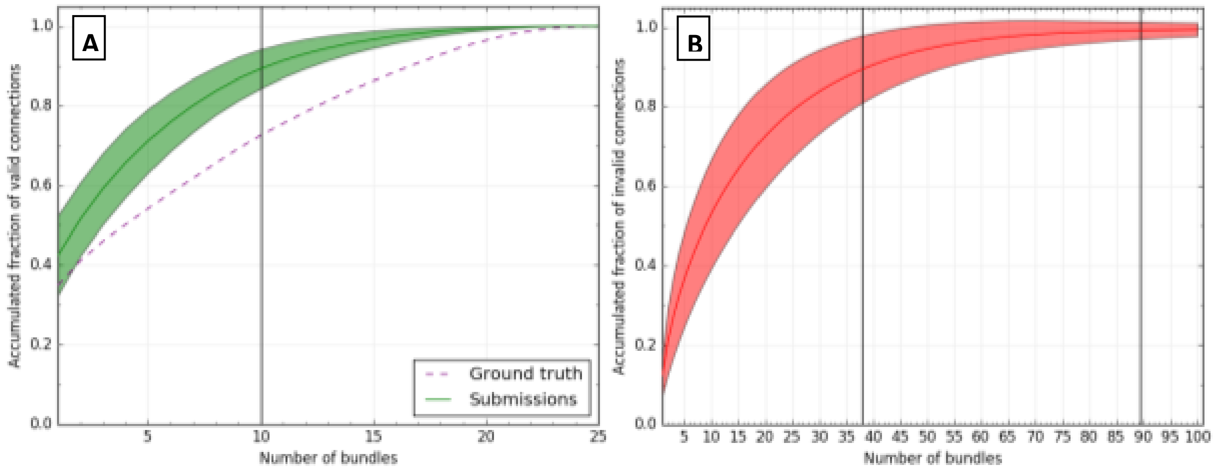

Supplementary Figure 5. Accumulated relative number of streamlines. Relative number of streamlines accumulated over the number of bundles sorted descending by their relative streamline count. The graphs show mean and standard deviation over all submissions, respectively (red: invalid, green: valid). A: On average, the first 10 VBs (40%) contained 90% of all valid streamlines (vertical line). B: On average, the first 38 IBs (43%) contained 90% of all invalid streamlines (first vertical line, the second vertical line indicates the mean number of invalid bundles across all submissions). Thus, the invalid bundles did not consist of randomly distributed spurious streamlines but instead were organized following a similar distribution as the valid bundles. The most obvious difference between the distributions was that the corpus callosum resembles a huge valid bundle with nearly 40% of all streamlines.

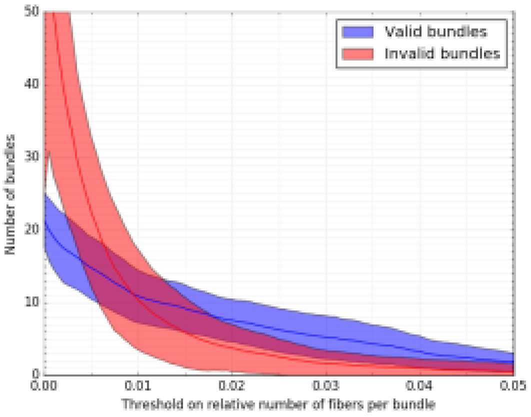

Supplementary Figure 6. Bundle selection on basis of fiber count. Comparison of number of invalid bundles versus number of valid bundles at different fiber count thresholds. The threshold was selected relative to the total fiber count of the corresponding submission. Invalid bundles can only be decreased at the expense of losing significant numbers of valid bundles. About one half of the valid bundles is lost until the number of invalid bundles drops below the number of valid bundles.

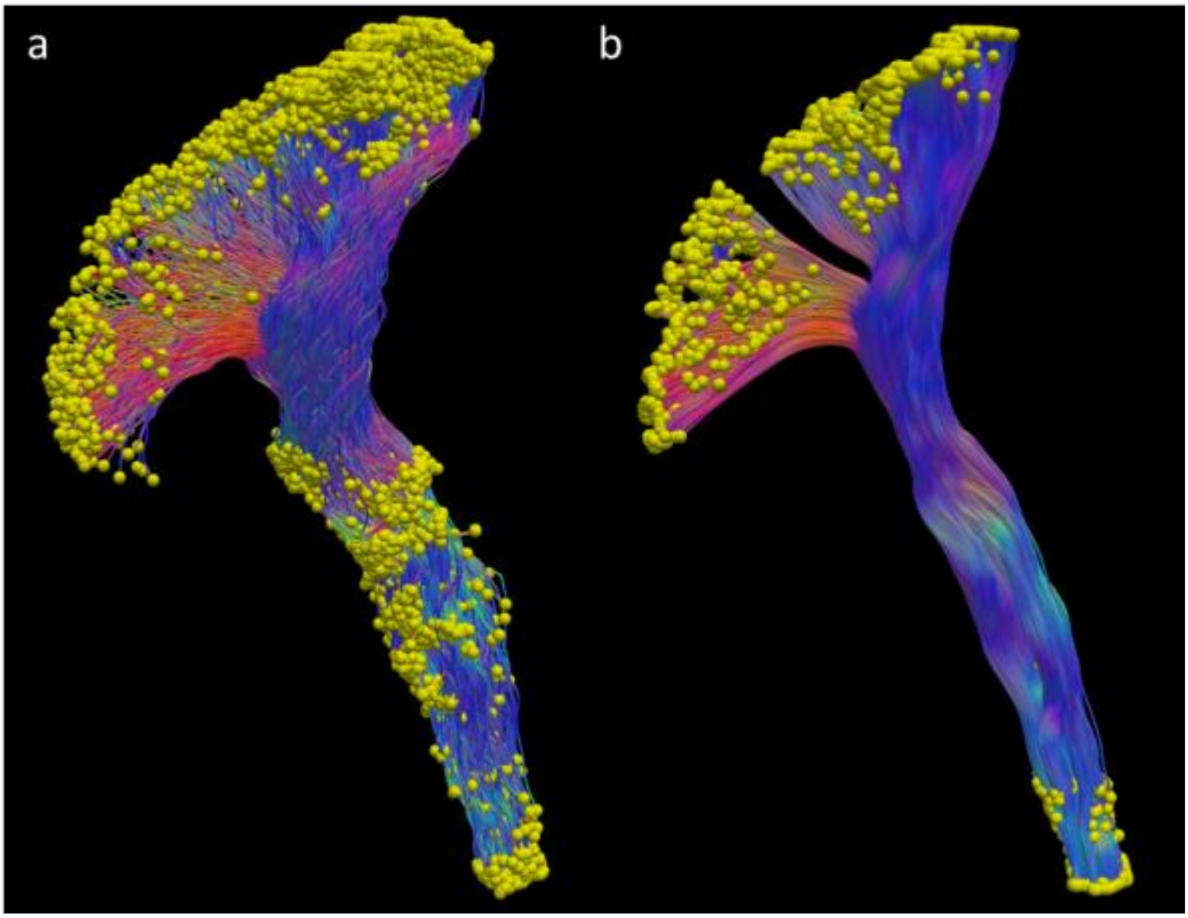

Supplementary Figure 7. Bundle refinement prior to phantom generation. Original CST extracted from the whole-brain global tractography result (a). The yellow spheres indicate fiber endpoints. (b) shows the corresponding smoothed streamlines where the fiber endpoints are mostly distributed at the bundle end-points. This is called the "refined bundle".

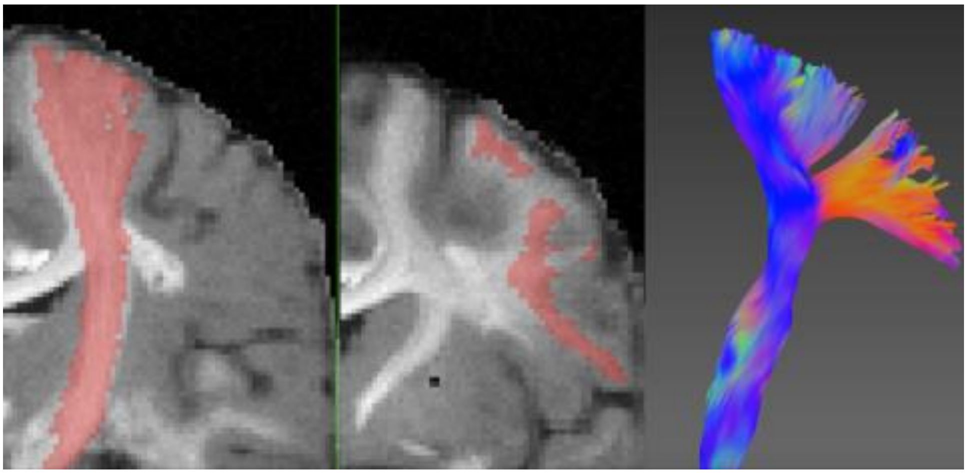

Supplementary Figure 8. Restrictive definition of a valid connection by tractometer metrics. Illustration of the corticospinal tract (CST) bundle volume (a) and endpoints distribution (b) in red overlay. As soon as a submitted streamline (c) has a single point exiting the bundle volume or not exactly terminating within the endpoints distribution, the streamline will be flagged as an invalid connection.

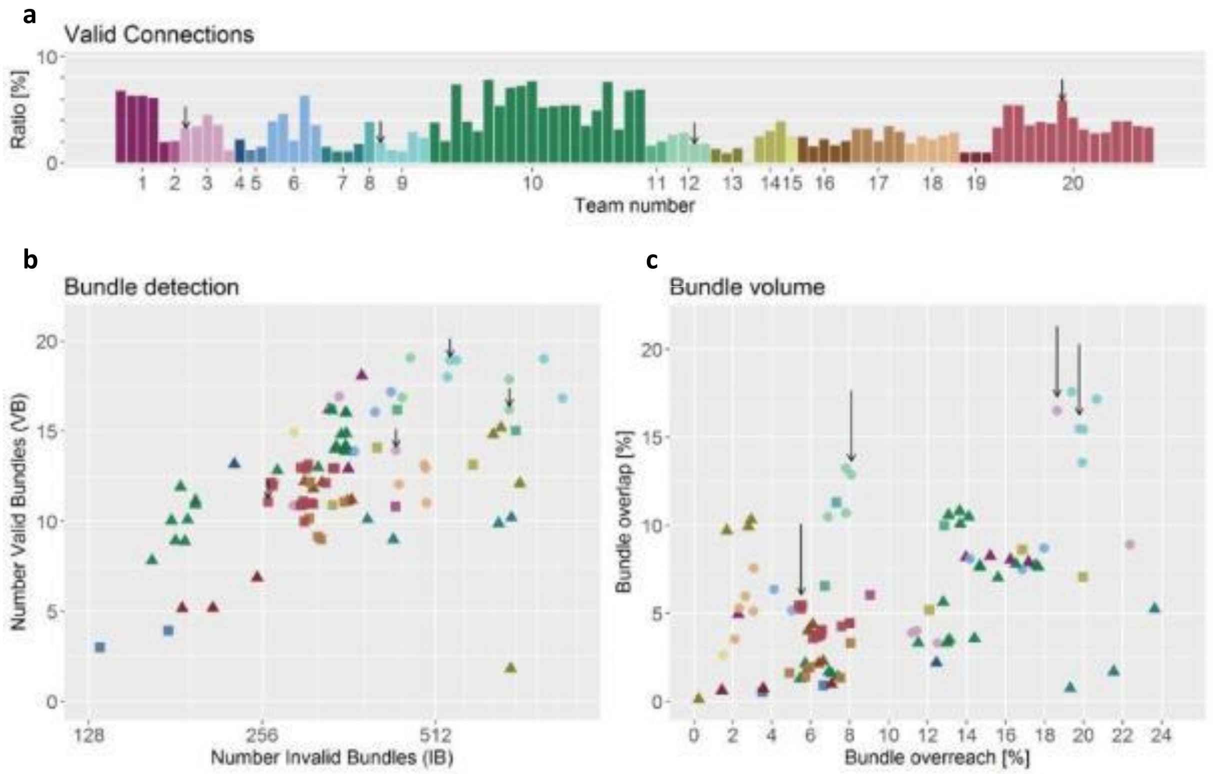

Supplementary Figure 9. Results using traditional scoring. In the Tractometer metrics, a *valid connection* is defined as a streamline that connects expected regions of interest without exiting the expected bundle mask. This definition is extremely demanding for current tractography algorithms (please note the altered axis scalings). When scoring based on these metrics, on average over all submissions, only 3% ± 2% of the submitted streamlines were valid (a). 13 ± 3 valid bundles were detected, accompanied by 376 ± 149 invalid bundles detected (b). The average volume overlap was 6% ± 10% (c). At best, a technique achieved 8% valid connections and 92% invalid connections.

Supplementary Notes

Supplementary Note 1: Abstracts of the different teams

Submissions were summarized using the following categories:

A - motion correction (i.e. correction of misaligned image volumes prior to analysis)

B - rotation of b-vectors (i.e. correction of b-vectors according to above misalignment)

C - distortion correction (i.e. correction of distortions caused by magnetic field inhomogeneities)

D - spike correction (i.e. detection and correction of spiked slices)

E – denoising (i.e. application of denoising filter prior to analysis)

F – upsampling (i.e. interpolation of image to 1x1x1 T1-weighted image resolution)

G - diffusion model beyond diffusion tensor imaging (DTI) (e.g. constrained spherical deconvolution)

H - tractography beyond deterministic (e.g. probabilistic tractography)

I - anatomical priors (i.e. incorporation of atlas information or manual selection of streamlines)

J - streamline filtering (e.g. application of length threshold on streamlines)

K - advanced streamline filtering (i.e. application of SIFT or COMMIT)

L - streamline clustering (i.e. application of clustering approach for streamline selection)

TEAM 1 **Submissions 1_0 to 1_4 (5 submissions)**

J Zhong

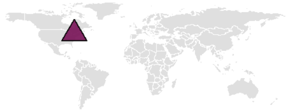

Pipeline description 1 – 1_4

1) Correction for Eddy Current and Subject Motion [FSL] and b-vector rotation
2) Filedmap correction [fugue from FSL]
3) brain extraction [FSL]
4) denoise using **LPCA filter**[3]
5) **single tensor** fiber tracking using MRtrix with Tensor_Prob algorithm [MRrix]

Pipeline description 2 – 1_2
1) Correction for Eddy Current and Subject Motion [FSL] and b-vector rotation
2) Filedmap correction [fugue from FSL]
3) brain extraction [FSL]
4) denoise using **AONLM filter**[3]
5) fiber tracking using MRtrix with sd_stream algorithm [MRrix]

Pipeline description 3 – 1_1
1) Correction for Eddy Current and Subject Motion [FSL] and b-vector rotation
2) Filedmap correction [fugue from FSL]
3) brain extraction [FSL]
4) denoise using **AONLM filter** then **LPCA filter**[3]
5) fiber tracking using MRtrix with sd_stream algorithm [MRrix]

Pipeline description 4 – 1_3
1) Correction for Eddy Current and Subject Motion [FSL] and b-vector rotation
2) Filedmap correction [fugue from FSL]
3) brain extraction [FSL]
4) denoise using **PRINLM filter**[3] (Smooth 2)
5) fiber tracking using MRtrix with sd_stream algorithm [MRrix]

Pipeline description 5 – 1_0
1) Correction for Eddy Current and Subject Motion [FSL] and b-vector rotation
2) Filedmap correction [fugue from FSL]
3) denoise using **rank and edge constraints**[4]
4) brain extraction [FSL]
5) fiber tracking using MRtrix with sd_stream algorithm [MRrix]

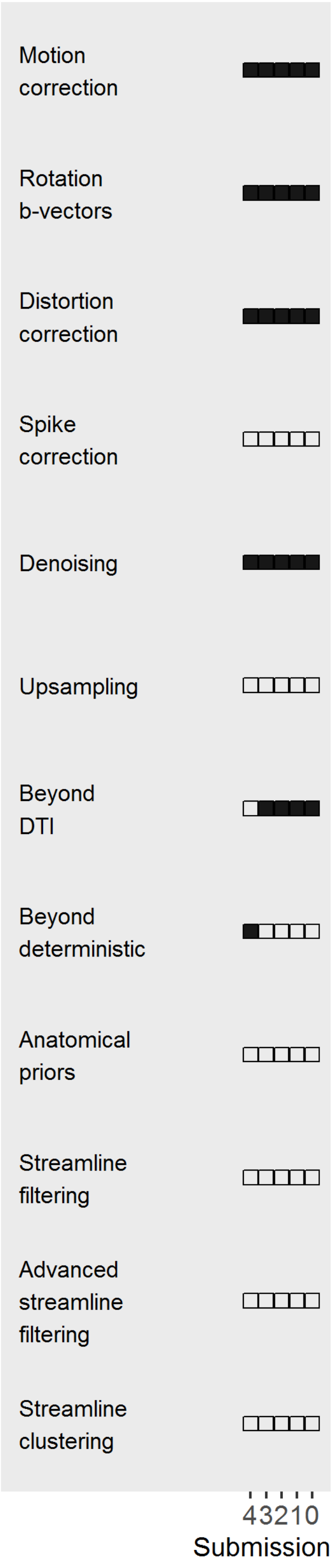

Team 2 - Submission 2_0 (1 submission)

M Chamberland, C Tax

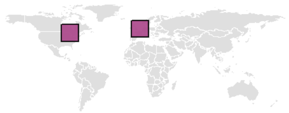

GENERAL preprocessing:
Extracting Dwi brain mask using Bet from FSL[5].
Extracting T1 mask using Bet from FSL .
Denoising using NLM from Dipy (DWI:sigma 15, T1:sigma 18).
Registering the original T1 to upsampled B0 using FLIRT from FSL.
Applying ANTS non-linear registration to the FLIRTed T1 image.
Extracting WM, GM and CSF from T1 using FAST from FSL.

//Local modeling
Creation of Tensor map using Dipy[6].
Creation of the FA map using Dipy.
Downsampling WM mask using AFNI (3dresample to 2x2x2)[7].
Finding response function using recursive calibration inside WM mask with Dipy (SH=8)[8].
Computing fODFs with CSD (whole brain mask) using Dipy.
Upsampling fODFS and FA by a factor of 2 (1x1x1 space) using Mrtrix[9].

//Tracking
Extracting maxima from fODFs using the Fibernavigator.
Performing real-time multi-peak tractography[10] with the following parameters:
Algorithm: Vnext = (f*Vcurr) + (1-f)*((1-g)*Vprev + g*Vcurr)
Seed mask: WM (502 861 seeds, 1 per voxel).
Exclusion mask: CSF.
Tracking mask: FA > 0.1 (f-param).
Step size: 1mm.
Angular threshold: 35deg.
g Param: 0.20.
min length: 60mm.
max length: 250mm.Export to real-time-peak-deflection-rc-dipy-fa.tck: 162 804 fibers.

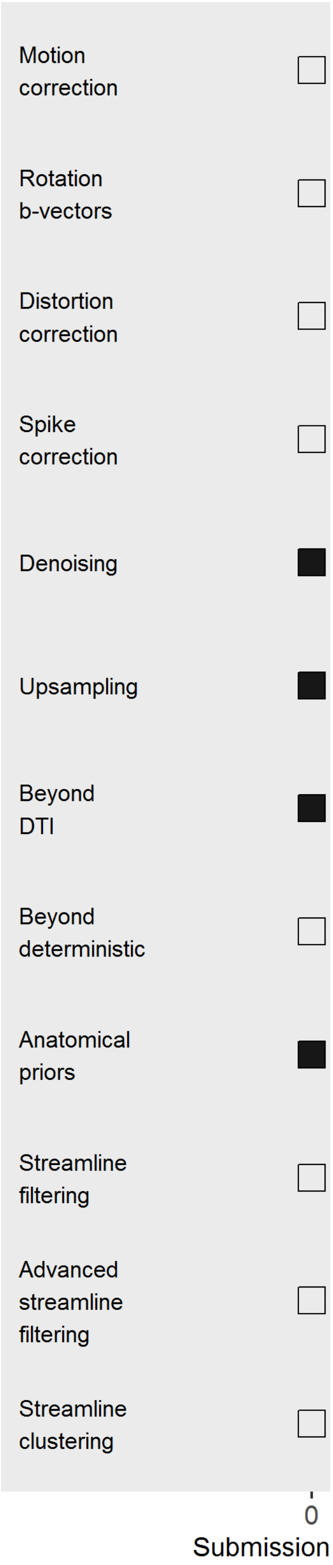

Team 3 – Submissions 3_0 to 3_4 (5 submissions)

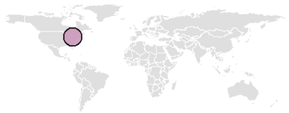

The diffusion images were processed in DSI Studio (http://dsi-studio.labsolver.org). Eddy current correction with b-table rotation was conducted by registering each DWI to the b0 image. In submission 1 to 3. The DWI data were further upsampled to the T1 space for 3. In submission 1, 2, 4, the diffusion data were reconstructed using generalized q-sampling imaging[11] with default parameters assigned in DSI Studio. In submission 3 and 5, diffusion decomposition[12] was used to reconstruct the data. A deterministic fiber tracking algorithm was used to generate whole brain fiber tracking[13]. An angular threshold of 70 degrees was used. The step size and anisotropy threshold were determined automatically by the tool. A hierarchical clustering method[14] was applied. The 50 largest clusters were selected and merged, whereas the small clusters were removed. The final results were submitted in trk format.

Pipeline 1 corresponds to 3_3

Pipeline 2 corresponds to 3_0

Pipeline 3 corresponds to 3_4

Pipeline 4 corresponds to 3_2

Pipeline 5 corresponds to 3_1

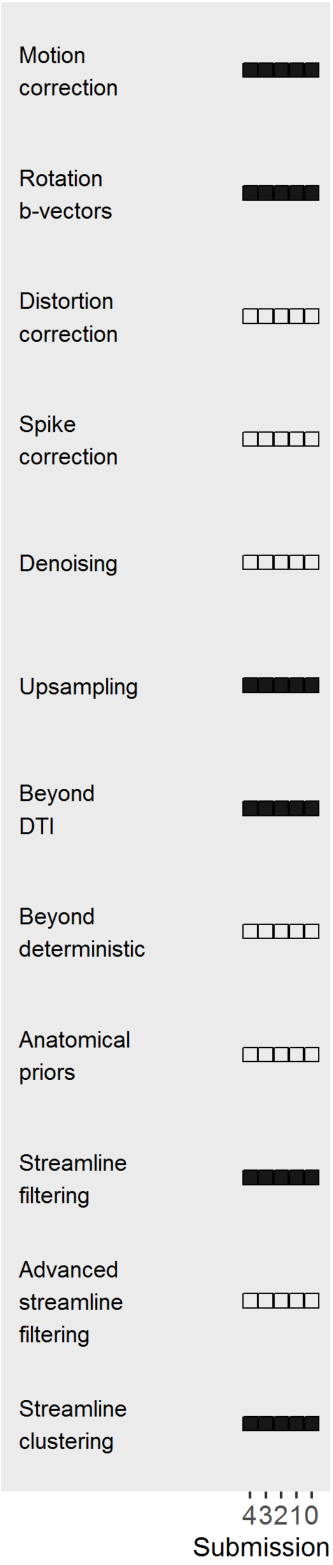

Team 4 – Submission 4_0 (1 submission)

Y-C Lin

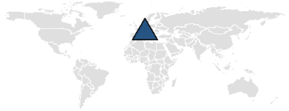

Pipelines description

The fiber bundle analysis were composed for three essential segments: (1) eddy current distortion, fieldmap correction and motion correction, (2) tensor calculation, reconstruction the fiber and (3) fiber classification into 26 fiber bundles by EM-step, and merge into whole brain tractogram.

-Eddy Current Correction is correct for these distortions, artifacts (include shear, enhanced background, image intensity loss, and image blurring), and head motion. For those correction perform affine registration to a reference volume by FSL (FMRIB). EPI distortion and fieldmap correction using FSL's FUGUE processing.

- Start the tensor fitting and calculation of fractional anisotropy (FA) value. The fiber-tracking used the streamlines approach, optimized with nearest neighbor interpolation. The range of the fiber length are 20 to 500 (mm).

- Fiber classification into 26 fiber bundles by EM-step with ten features (include first point, middle point and last point of the coordinate (x, y, z) ; length of the fiber) are extracted into the EM (expectation-maximization) step. To correct the fiber pathway are automatic classification into 26 fiber bundles by EM algorithm and select valid bundles.

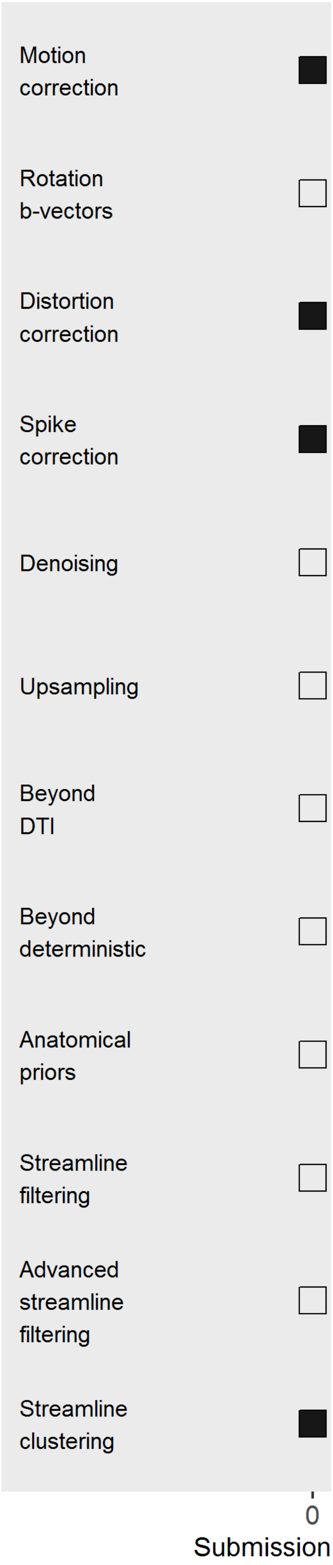

Team 5 – Submissions 5_0 and 5_1 (2 submissions)

Q Ji, W E Reddick, J O Glass

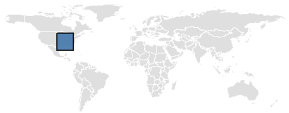

Pipeline descriptions :

Pipeline 1 : 5_1

- Eddy current and motion corrections.
- FSL bedpost were run on the data.
- 17800 seeds voxels were chosen within white matter randomly. 10 fibers for each seed were generated using probabilistic fiber-tracking implemented in FSL[15]. Each streamline was recorded.
- Spline processing and a filter were applied to each generated fiber.

Pipeline 2 : 5_0

- Eddy current and motion corrections
- 17800 seeds voxels were chosen within white matter randomly, a stream line based algorithm was used to track 10 fibers from each seed voxel.
- Filter was applied on each fiber, the fibers below threshold were discarded.

.

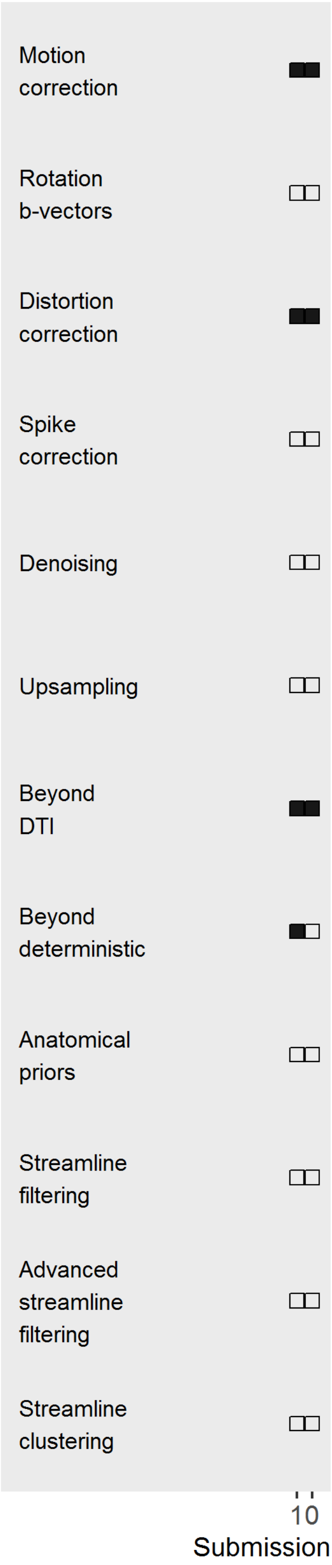

Team 6 – Submissions 6_0 to 6_3 (4 submissions)

D Qixiang Chen

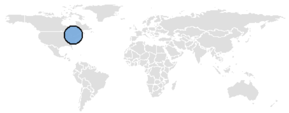

Common pipeline :

1. Denoising with PRINLM with 2mm smoothing[16]
2. Reverse phase-encoding correction with TOPUP[17]*
3. Motion correction with FSL and proper rotational correction to bvecs.[18]
4. Brain mask generated with FSL bet2 with averaged DWI image.
5. Seed mask is generated with FA map thresholded to >0.2 FA, and then combined with the brain mask eroded by 5 voxels to avoid seeding in regions close to the skull.

Pipeline 1 :

Constrained spherical deconvolution with MRtrix 3[9]

- Estimate constrained spherical deconvolution odfs
- Deterministic tractography with min length 20mm, angle 60deg, cutoff 0.2, number of tracks = 150,000, with 4-th order runge-kutta integration.
- * Note there are 2 files for MRtrix:
    - step size 0.1 mm: **csd_stp01_a45.tck** – 6_3
    - step size 0.5 mm: **csd_stp05_a45.tck** – 6_1

Pipeline 2 : Submission

2-tensor tractography with eXtended Streamline Tractography (XST)

- Deterministic tractography with FA threshold 0.2, tensor fraction 0.1, angle threshold 1.04 rad (60 deg), min-length 20, step size 0.3, and 2 seeds per voxels.
- File: **xst_03stp_a60.vtk** – 6_4

Pipeline 3 :

EuDX tractography with Dipy[6]

- Estimate constrained spherical deconvolution peaks
- Run EuDX with angle_threshold 45deg, anisotropy cutoff 0.3, 2 seeds per voxel, step size 0.5
- File: **eudx_45deg05stp.vtk** – 6_0

Pipeline 4:

Deterministic Probabilistic tractography with Dipy

- Estimate constrained spherical deconvolution peaks and get coeffecients
- Run deterministic maximum direction getter with max_angle=25
- Run local tracking with step size 0.5 with 2 seeds per voxel
- File: **dmsc.vtk** – 6_2

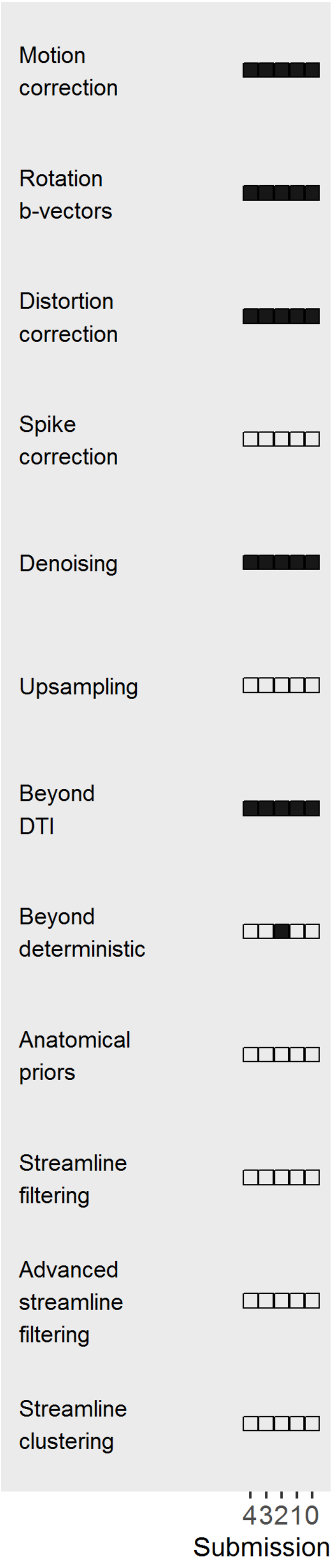

Team 7 – Submissions 7_0 to 7_3 (4 submissions)

Y Feng, C Gao, Y Wu, J Ma, R He, Q Li, C-F Westin

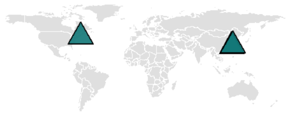

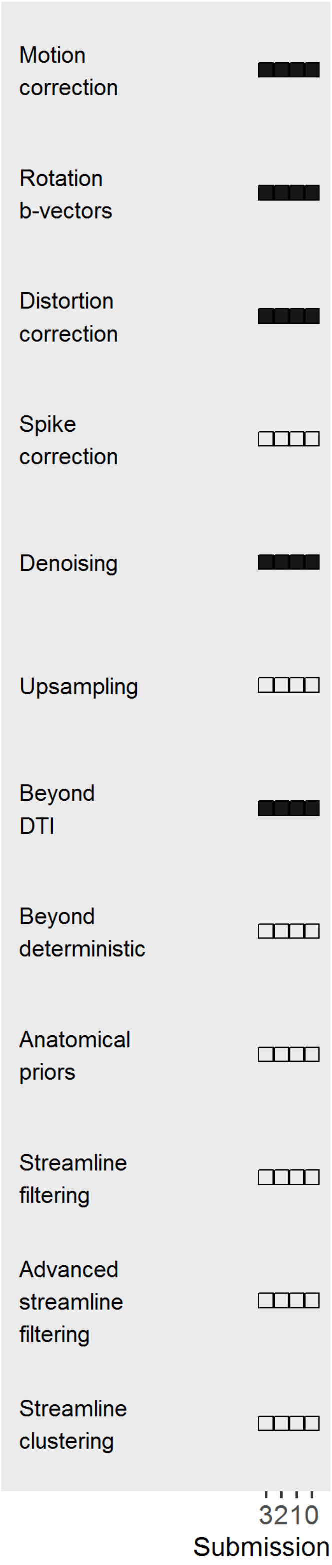

1. Motion correction

Correcting for subject motion & eddy current induced geometric distortions: Only bulk motion considered is the general object movement, the misalignment will cause blurring of the diffusion tensor image, as well as the related orientation of diffusion. The rigid registration with quaternion is applied to all gradient directions to align them with B0 image. The reorientation of the corresponding B-matrix is completed so that orientation information is correctly preserved[18].

2. Distortion correction

Correcting for EPI/susceptibility distortions (shape and intensity): The phase direction distortion of EPI is corrected with the provided field map. The approach we implemented is based on the work of[19]. In this way, the geometry distortion correction of subject motion and EPI distortion can be performed in one interpolation step to minimize blurring effects. However, geometric distortion of EPI is accompanied by compression or stretching of voxels so that the voxel brightness is altered as well as voxel position. To facilitate intensity distortion correction, we complete the geometry distortion correction of subject motion and EPI distortion in two separate steps. When a local field gradient causes a great phase change across a voxel, the signal from that voxel is not displaced but lost all together due to signal dephasing. Afterwards, there are unrecoverable EPI signal losses.

3.Diffusion model beyond DTI.

1. Preprocessing of DWI data. The brain mask is obtained using Mrtrix by averaged DWI images with default threshold. Then, a tensor map and FA map are generated by DTI model. High anisotropy mask is extracted by FA map with FA threshold value 0.2. Finally, the WM mask is produced by the intersection of brain mask and high anisotropy mask.
2. Global consistency model. A spherical double-lobe basis function is used to form an overcomplete dictionary that guarantees the local sparsity of FOD. And a global consistency spatial model which incorporated a spatial priori information based on the Bayesian framework is developed using the coefficients of the basis function, which integrates the local fiber distributions in the neighbourhood, detailed in Wu et al.[20]. Due to the deconvolution-based process, the response function is estimated by the average of diffusion signal with FA threshold value larger than 0.7. The exponent enhancement number used in the experiment is 10.

3. Deterministic tracking. A HARDI based streamline tracking method which chooses the closed fiber peak orientation extracted from FOD of the last tracking step as the tracking direction is carried out using formula Vnext = f*Vcurr + (1-f)* Vprev with f=0.7. The angular threshold for contiguous step is 50 degree to avoid unreasonable turn. The step length is 1mm. The seed point position is random generated in per WM voxel. The range of the fiber length is between 50 mm and 500 mm, and fibers whose length out of range are exclude. In our result, 13871 fibers are generated totally and 10000 fiber trajectories are reserved for the Pipeline.

<u>Pipeline 1</u>– 7_0

1. Preprocessing of DWI data. A motion correction procedure is executed to eliminate pseudo-signal.
2. Global consistency model. A spatial consistency regularization model is performed which integrates the local fiber distributions in the neighbourhood.
3. Deterministic tracking. A HARDI based streamline tracking method which chooses the closed fiber peak orientation extracted from Fod of the last tracking step as the tracking direction is carried out.

100,000 fiber trajectories is reserved.

<u>Pipeline 2</u>– 7_3

1. Preprocessing of DWI data. A motion correction procedure is executed to eliminate pseudo-signal.
2. Global consistency model. A spatial consistency regularization model is performed which integrates the local fiber distributions in the neighbourhood.
3. Deterministic tracking. A HARDI based streamline tracking method which chooses the closed fiber peak orientation extracted from FOD of the last tracking step as the tracking direction is carried out.

10,000 fiber trajectories is reserved.

<u>Pipeline 3</u>– 7_1

1. Preprocessing of DWI data. A motion correction procedure is executed to eliminate pseudo-signal and a geometric correction is executed.
2. Global consistency model. A spatial consistency regularization model is performed which integrates the local fiber distributions in the neighbourhood.
3. Deterministic tracking. A HARDI based streamline tracking method which chooses the closed fiber peak orientation extracted from FOD of the last tracking step as the tracking direction is carried out.

100,000 fiber trajectories is reserved.

<u>Pipeline 4</u>– 7_2

1. Preprocessing of DWI data. A motion correction procedure is executed to eliminate pseudo-signal and a geometric correction is executed.
2. Global consistency model. A spatial consistency regularization model is performed which integrates the local fiber distributions in the neighbourhood.
3. Deterministic tracking. A HARDI based streamline tracking method which chooses the closed fiber peak orientation extracted from FOD of the last tracking step as the tracking direction is carried out.

10,000 fiber trajectories is reserved.

Team 8 – Submission 8_0 (1 submission)

S Deslauriers-Gauthier, J-O Ocegueda-Gonzalez, S St-Jean, M Paquette, G Girard

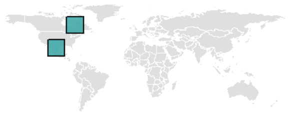

Pipelines description:

+ Unspiking (in-house algorithm)

+ motion correction: We defined a graph where each of its **n** nodes represents a dMRI scan (excluding the B0 image). The edge weights are given by the angle between the b-vectors associated with their edge nodes. We computed the minimum spanning tree (MST) of the graph and rigidly registered all pairs of images corresponding to adjacent nodes in the MST by maximization of their mutual information (exactly **n-1** registrations). The registration between any pair of scans can then be computed by following the path between their corresponding nodes in the tree and composing the transforms associated with the path's edges. We selected the center of the tree as reference and registered the rest of the images towards it. The average of the dw images has higher SNR and can then be registered to the B0.

+ Susceptibility-induced geometric distortion correction: We directly applied the deformation field defined by the off-resonance field provided by the organizers of the challenge. The bandwidth per voxel along the phase-encoding direction was also known to be 9.26 Hz per voxel.

+ denoising Non Local Spatial and Angular Matching (NLSAM)[21] with 5 angular neighbors

+ Spherical finite rate of innovation (soon to be in dipy)[22]

+ Deterministic particle filtering tractography[23]

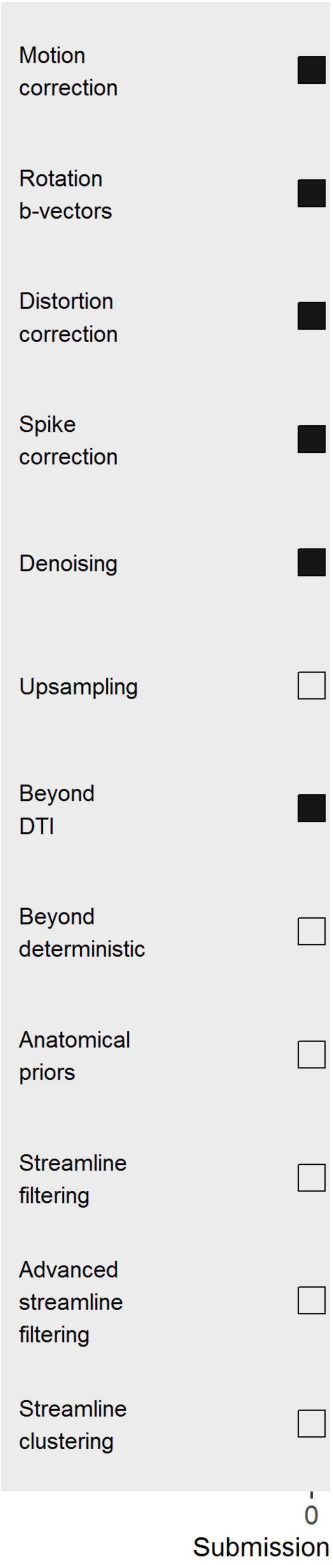

Team 9 – Submissions 9_0 to 9_4 (5 submissions)

F Rheault, S St-Jean, J Sidhu

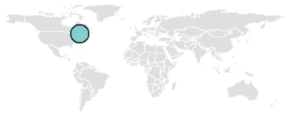

Common pipeline

- Motion correction (with a nearest neighbor interpolation)
- Correction of the *b vectors* using the corresponding affine matrix
- Flip of the *z gradient* value
- NLSAM for denoising and spike correction on the raw DWI[21] - Number of angular neighbors : 5
- NLM on the T1 - Automatic noise sigma estimation
- Upsample of the DWI to the T1 space using trilinear interpolation
- Segmentation of white matter from the denoised T1 (using FSL FAST)
- Dilatation of the *WM* mask to remove potential hole in it - 1 iteration
- Mrtrix Constrained Spherical Deconvolution
    - Response function estimated from voxel with FA higher than 0.65
    - Spherical Harmonics of order 6
    - Mask used was a B0 mask in the T1 space (using BET)
- Mrtrix deterministic and probabilistic tracking
    - Seeding from the dilated WM mask
    - Masking with the dilated WM mask
    - 2 millions streamlines with a 0.2mm step-size

Deterministic

- Pipeline 1 – 9_3
    - Linearization of the streamline with a 0.001mm threshold[24]
    - Streamline subset selection using a recursive implementation of QuickBundles with a distance threshold of 2.5mm[25]
- Pipeline 2 – 9_4
    - Linearization of the streamline with a 0.01mm threshold
    - Recursive QuickBundles with a distance threshold of 2.5mm
- Pipeline 3 – 9_0
    - Linearization of the streamline with a 0.1mm threshold
    - Recursive QuickBundles with a distance threshold of 2.5mm

Probabilistic

- Pipeline 4 – 9_1
    - Linearization of the streamline with a 0.01mm threshold
    - Recursive QuickBundles with a distance threshold

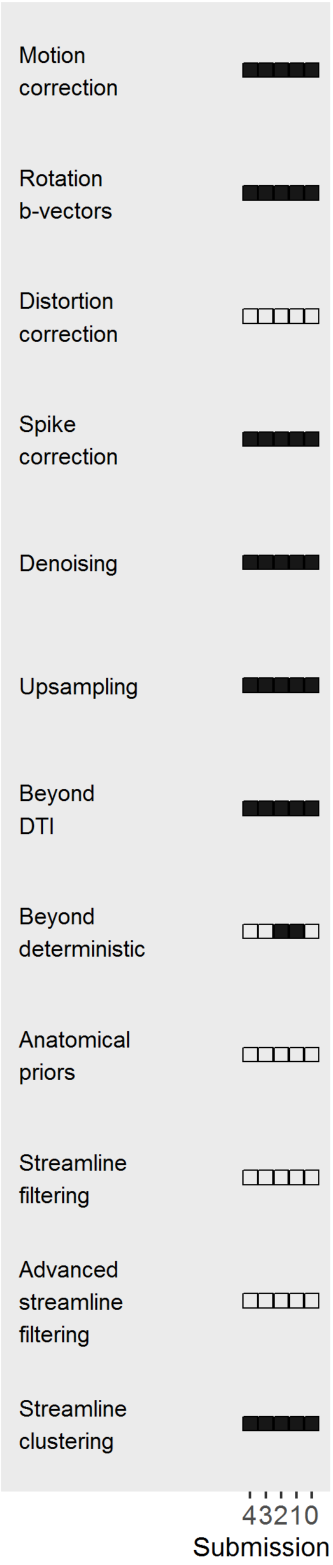

of 2.5mm

- Pipeline 5 – 9_2
    - Linearization of the streamline with a 0.1mm threshold
    - Recursive QuickBundles with a distance threshold of 2.5mm

Team 10 – Submissions 10_0 to 10_19 (20 submissions)

S David, R van den Elzen, M Froeling, F Guo, W Haakma, A Heemskerk, A Leemans, H Mesri, C Tax

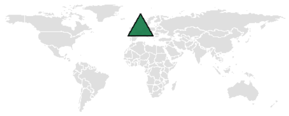

Pipelines description

The different pipelines are outlined in Fig. 1 (numbered 10_0 to 10_19). A pipeline typically consists of the following steps, and we have tested different combinations of options for each step:

1. Denoising:
    a. No denoising
    b. Rician noise correction
2. Motion correction:
    a. No registration
    b. Rigid registration
3. EPI distortion correction:
    a. No correction
    b. B0 fieldmap
    c. Registration to T1
4. Outlier detection and rejection:
    a. No outlier detection
    b. REKINDLE
5. Diffusion modeling:
    a. DTI
    b. CSD
6. Postprocessing of fibertracts:
    a. No postprocessing
    b. Gray matter (GM) postprocessing

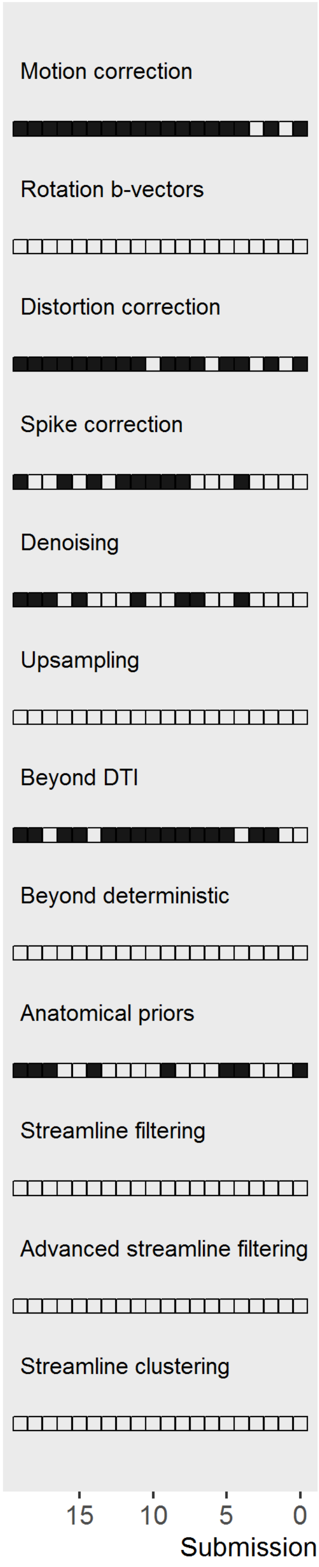

Detailed description of each option:

- Rician noise correction: Noise removal by convolution of a linear minimum mean square error estimator using a Rician noise model with the diffusion weighted images (DWIs) using a 5 x 5 pixels Gaussian kernel[26,27]. The SNR was determined from the background noise and was found to be 20 for the b=0 image.
- Rigid registration: A rigid (6 degrees of freedom) coregistration technique based on mutual information was used to realign the DWIs to the first non-DWI[28,29]. It was wrongly assumed here that no B-matrix rotation was applied when generating the synthetic dataset, and that performing B–matrix rotation would therefore result in "overcorrection".
- Fieldmap correction: Image deformations due to field inhomogeneities were corrected based on the provided B0 field map. Signal accumulation and loss was not corrected[27].
- Registration to T1: Non-rigid registration of the fractional anisotropy image to the T1 image[30].
- REKINDLE : Outlier detection and removal with iteratively reweighted linear least squares estimation (κ=6)[28,31].
- DTI: Iterative weighted linear least squares estimation[28,32].
- CSD: Determination of the fiber orientation distribution function with constrained spherical deconvolution (lmax = 6) and recursive calibration of the response function (peak ratio threshold 0.01)[8,28,33]. In case outlier detection with REKINDLE was used in the previous step, rejected points were interpolated based on the robust diffusion tensor, so that an equal amount of directions per voxel could be used for CSD.
- Tractography: Deterministic CSD or DTI tractography was performed with ExploreDTI[28] using the following tracking parameters: Seed Point Resolution = [2 2 2]; Step Size = 1 (mm); Angle Threshold = 30 (degrees); Fiber Length Range = [50 175] (mm); Seed FA Threshold = 0.2; FA Track Range = [0.2 1]; MD Track Range = [0 2*10^-3]; Interpolation Method = "Linear"; Random Sampling = 1; randp = 1; CSD Peak Threshold = 0.1;
- GM postprocessing: The T1 weighted anatomical image was segmented and binarized into 3 components: white matter (WM), GM and cerebrospinal fluid (CSF) using the FAST toolbox[34] as implemented in FSL. The GM mask was dilated using a modal dilation which takes the most common value of the non-zero values of the neighbors[35]. Voxels which were dilated into the CSF were masked out with the CSF mask from the FAST segmentation. Only streamlines with both start and endpoint in the GM were maintained.

Fig. 1 : Analysis pipelines

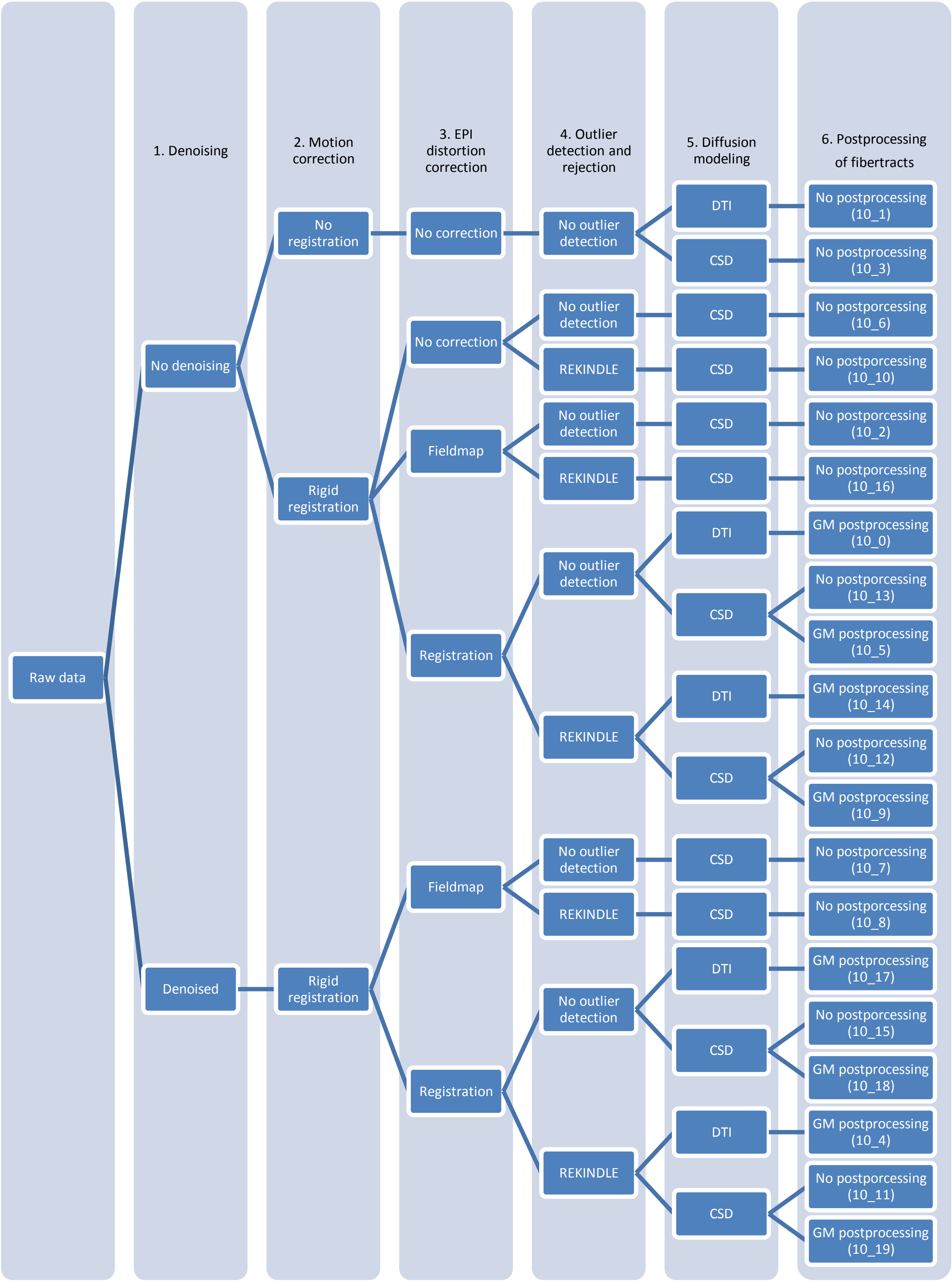

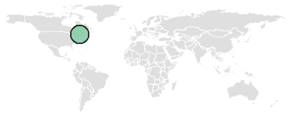

Team 11 – Submissions 11_0 to 11_1 (2 submissions)

S St-Jean, F Rheault, G Girard

Preprocessing

+ Motion correction (flirt)

+ Denoising and spike correction with Non Local Spatial and Angular Matching (NLSAM),
5 angular neighbors and Rician noise correction[21]

+ Upsampling to t1 space with CLARS[36]

Local reconstruction

+ Constrained Spherical Deconvolution (CSD) of order 6 (dipy)

Fiber tracking

+ in-house tracking (no pft) 1 seed/voxel T1 space, seed from FA > 0.1 mask (Two Pipelines branch out)[37]

Total of 2 Pipelines produced

+ A. Upsampling to T1 space, deterministic tracking – 11_1

+ B. Upsampling to T1 space, probabilistic tracking – 11_0

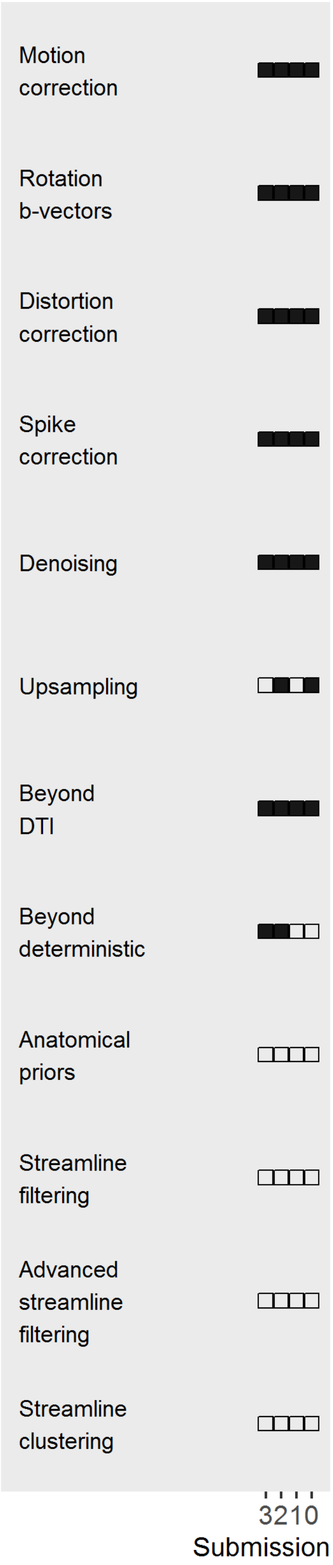

Team 12 – Submissions 12_0 to 12_3 (4 submissions)

M Paquette, J-O Ocegueda-Gonzalez, S St-Jean, G Girard

Preprocessing

+ Unspiking (in-house algorithm)

+ Motion correction: We defined a graph where each of its **n** nodes represents a dwMRI scan (excluding the B0 image). The edge weights are given by the angle between the b-vectors associated with their edge nodes. We computed the minimum spanning tree (MST) of the graph and rigidly registered all pairs of images corresponding to adjacent nodes in the MST by maximization of their mutual information (exactly **n-1** registrations). The registration between any pair of scans can then be computed by following the path between their corresponding nodes in the tree and composing the transforms associated with the path's edges. We selected the center of the tree as reference and registered the rest of the images towards it. The average of the dw images has higher SNR and can then be registered to the B0.

+ Susceptibility-induced geometric distortion correction: We directly applied the deformation field defined by the off-resonance field provided by the organizers of the challenge. The bandwidth per voxel along the phase-encoding direction was also known to be 9.26 Hz per voxel.

+ Denoising with Non Local Spatial and Angular Matching (NLSAM), 5 angular neighbors and Rician noise correction[21]

+ Upsampling to t1 space (scilpy) (Two Pipelines branch out)

Local reconstruction

+ Constrained Spherical Deconvolution (CSD) of order 6 (dipy)

Fiber tracking

+ in-house tracking (no pft) 8 seeds/voxel diffusion space, 1 seed/voxel T1 space, seed from FA > 0.1 mask (Two Pipelines branch out)[37]

+ For the T1 space tracking, only keep the tracts inside an eroded brain mask to remove obvious outliers.

Total of 4 Pipelines produced

+ A. Diffusion space, deterministic tracking – 12_1

+ B. Diffusion space, probabilistic tracking – 12_3

+ C. Upsampling to T1 space, deterministic tracking – 12_0

+ D. Upsampling to T1 space, probabilistic tracking – 12_2

Team 13 – Submissions 13_0 to 13_3 (4 submissions)

A Boré, B Pinsard, C Bedetti, M Desrosiers, S Brambati, J Doyon

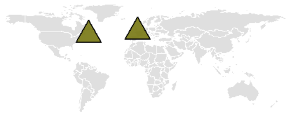

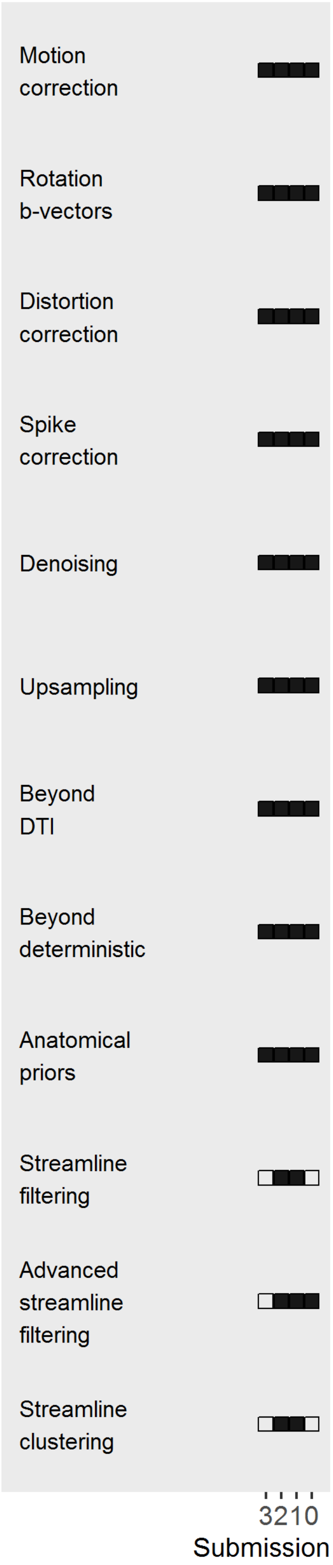

1 - Preprocessing

1.1 Diffusion Weighted Imaging

Data were preprocessed by first estimating motion of each volume of diffusion relative to first B0 using FSL FLIRT[5].

Then we detected spiked slices as having voxels in Fourier space in abnormal range compared to other directions of the same slice. Raw data spiked slices were re-interpolated from the 6 neighboring directions in term of diffusion vector encoding. Only neighbors non-spiked same and adjacent slices were considered to avoid correcting with artifacted data. Interpolation used motion parameters to account for motion between directions acquired, and 6 interpolated slices were averaged.

Then, interpolation of motion was applied to despiked data first filtered with Non-Local Means.

FSL topup tool corrected for EPI inhomogeneity induced distortions using reversed phase encoded B0[38].

1.2 Anatomical Imaging

T1 was analyzed with adapted freesurfer recon-all routine by skipping skullstriping step, replacing masking with AFNI 3dAutomask, and using template without skull for all operations to accomodate the simulated T1 scan provided.

Anatomical priors for Anatomically Constrained Tractography[39] as 5 Tissue Type (5TT) format for MRTrix was prepared from freesurfer surfaces and parcellation by computing partial volume maps for:

- cortical and cerebellar gray matter, - subcortical gray matter, - white matter, - csf, - abnormalities.

A mock gray matter region was set at the end of the brainstem to allow spinal streamlines to be found, and anterior (AC) and posterior commissure (PC) were added to white matter map.

2 - Reconstruction

Fibers orientation distribution (FODs) were estimated using constrained spherical harmonic degree lmax of 6[33].

3 - Tractography

We initiated tractography with a seeding dynamically using SIFT model[40]. The 2nd order Integration over Fiber Orientation Distribution (iFOD2) algorithm[41] provided by MRtrix in conjunction with ACT framework was used to recover streamlines[39].

Parameters used for tractography:

- Step 0.2mm - Maximum curvature per step: 9 degrees - Length: 50-300mm - FOD amplitude threshold for termination 0.1. We reconstructed 10 millions of streamlines.

The resulting tractogram is named hereafter“raw tractogram”.

4 - Submissions

Submission – 13_0

Starting from the raw tractrogram we used SIFT to improve its correspondence with the FODs reconstruction. It helped as well to reduce the size of the file and we ended with 430 000 streamlines.

Submission – 13_2

Starting from the raw tractogram, we filtered it using different rules:

- Streamlines that cross corpus collusum (CC) cannot cross brainstem (BS).

- Streamlines that cross BS cannot pass through both hemispheres.

- Streamlines that cross CC should require some symmetry aspects:

- The medial point of the streamline that cross the CC should be within the 2nd third of the fiber

- Within a streamline, points that cross the CC mask have to be contiguous.

- Splitting streamline at the medial point that cross the CC we compute the minimum average direct-flip (mdf) (dipy) of one half mirrored through medial wall with the other half to estimate the symmetry of the original streamline. Every streamline which has a distance higher than 4 is removed.

- Unilateral cerebello-cerebellar streamlines were remove.

Filtered streamlines were then selected through SIFT resulting in 450 000 streamlines.

4.3 Submission – 13_1

Starting from submission 2 before SIFT algorithm we ask a specialist in neuro-anatomy to manually select some bundles of streamlines using endpoints and waypoints landmarks. We added to this twelve manually segmented bundles, streamlines crossing the CC, AC, PC as well as those going through respectively BS and cerebellum. Finally, we again used SIFT algorithm and ended with 310000 streamlines.

4.4 Submission – 13_3

Last submission is a compilation of all streamlines that have been removed during filtering for submission 2. While we expect this submission to be poorly evaluated, it is of interest to check if filtering has not been too conservative and removed relevant filtered streamlines. This submissions contains 295 000 streamlines.

Team 14 – Submissions 14_0 to 14_2 (3 submissions)

A Sarica, R Vasta, J Yeatman, A Cerasa, A Quattrone

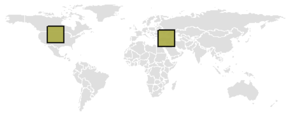

Pipeline 1

1) FSL Pre-processing

- a) *fslreorient2std* : T1 and Diffusion images were reoriented to match the orientation of the standard template image (MNI152).
- b) *SUSAN-Structure Preserving Noise Reduction* : T1 and Diffusion (here called dwi) images were denoised using non-linear filtering (default parameters). Outputs : i) T1_susan.nii.gz ii) dwi_susan.nii.gz
- c) *MELODIC-Multivariate Exploratory Linear Optimized Decomposition into Independent Components* : Artefacts in Diffusion image were removed by ICA denoising. Inputs : i) dwi_susan.nii.gz Parameters :
- i) *Motion correction* : None
- ii) *Spatial smoothing FWHM (mm)* : 5
- iii) *Temporal filtering* : Highpass Outputs : i) filtered_func_data.nii.gz
- b) *BET- Brain Extraction Tool :* Brain was extracted from T1 for the following step. Inputs : i) T1_susan.nii.gz Outputs : ii) T1_susan_brain.nii.gz
- c) *FEAT- FMRI Expert Analysis Tool :* Diffusion image was coregistered on structural image and on standard space. Inputs : i) T1_susan.nii.gz ii) T1_susan_brain.nii.gz iii) filtered_func_data.nii.gz Parameters : i) *Main structural image* : T1_susan.nii.gz, Normal Search, 6 DOF

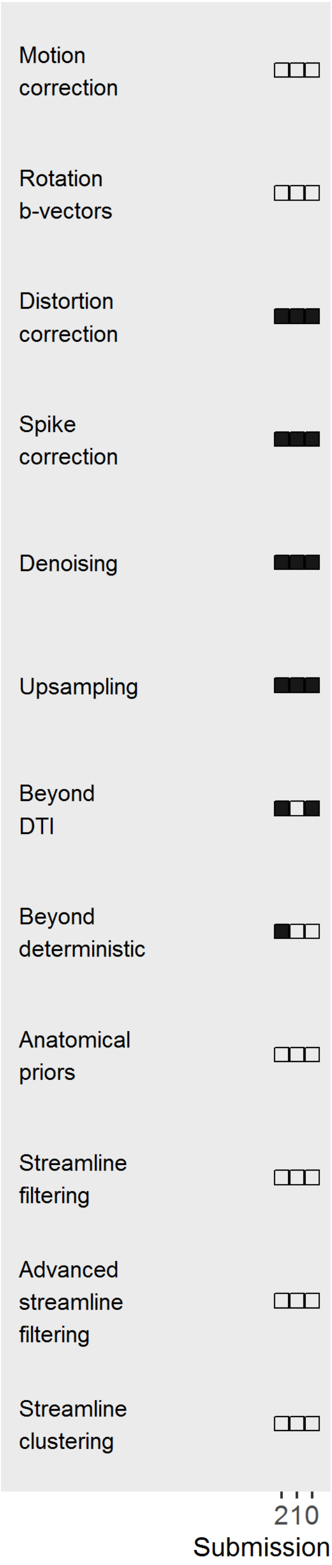

ii) *Standard space* : MNI152_T1_2mm, Normal Search, 6 DOF, Nonlinear, Warp resolution (mm)=10

Outputs : i) highres2standard ii) example_func2standard iii)

transformation matrices and warps.

2) MRTRIX-Diffusion-weighted MRI white matter tractography

. a) The pre–processed Diffusion image, registered on the pre-processed T1, and its brain mask were converted into mrtrix format *(.mif*) (*mrconvert* command). Same applies for b-values and gradient directions, where z coordinate was inverted (-z), for respecting mrtrix conventions.

. b) Tensor components and fractional anisotropy mask were generated as described in mrtrix documentation.

. c) A mask of high anisotropy voxels was generated by eroding the FA mask with a threshold of 0.7.

. d) The response function SH coefficients were estimated from the DW signal in the single-fibre voxels, with a harmonic order of 4.

. e) The constrained spherical deconvolution (CSD) was performed with a maximum harmonic order set to 4.

. f) Tensor-based fibre-tracking (DT_STREAM.tck – 14_1), deterministic fibre-tracking (SD_STREAM.tck – 14_2) and probabilistic fibre-tracking (SD_PROB.tck – 14_0) using the CSD were performed using with 200,000 number of fibers.

3) CONVERSION OF TRACTOGRAMS TO ORIGINAL SPACE

. *a) gen_unit_warp* script was used for extracting nowarp images from Diffusion image.

. *b) applywarp* was used for applying inverse transformation produced during registration of Diffusion-T1-standard space (step 1.c).

. *c) normalize_tracks* script was applied on the tractograms (.tck) produced during step 2.f, for converting them in the original space.

. *d)* Tractograms normalized to original space, were checked by using the script provided by the challenge organizers (validate_tracts_space.py).

Team 15 – Submissions 15_0 (1 submission)

A R. Khan, W Hodges, S Alexander

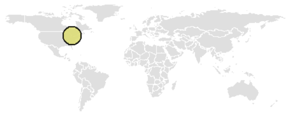

Pipeline is an amalgamation of tools from FSL and the ITK-based BrightMatter neurosurgical planning software (Synaptive Medical).

1. DWI skull-stripping (FSL, bet -f 0.1)
2. Motion/eddy current correction to b0 (FSL, eddy_correct)
3. Field-map unwarping (FSL, fugue)
4. Tensor estimation and tracking (Synaptive)
    a. Deterministic streamline tracking
    b. 4th order Runge-Kutta interpolation
    c. Step size = 0.5 mm
    d. Max Angle = 45 deg
    e. Min tract length = 10mm
    f. FA seed threshold = 0.3
    g. FA stopping criteria = 0.2
    h. Tract culling to 10,000 tracts (keeping longest tracts)

Tracts are in VTK format, with RAS reference.

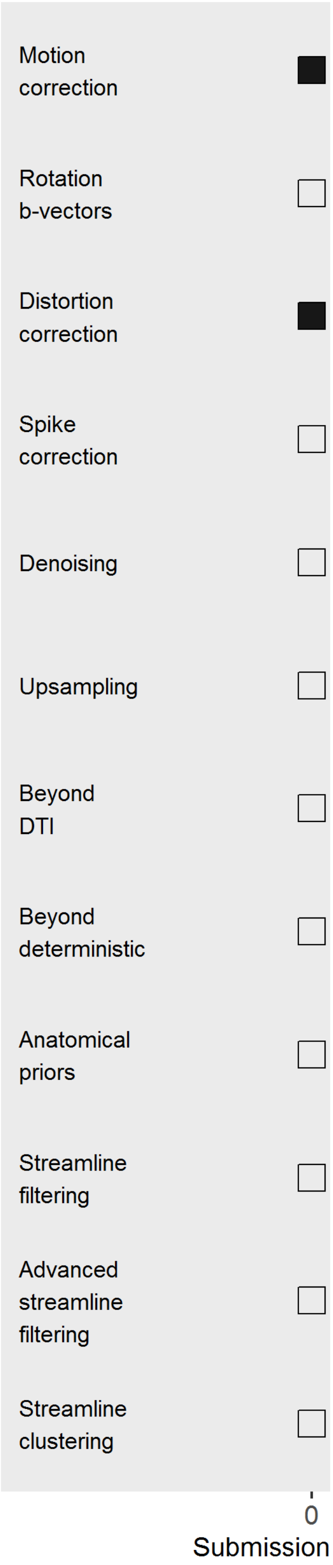

Team 16 – Submissions 16_0 to 16_4 (5 submissions)

D Romascano, O Esteban, A Auria, J-P Thiran

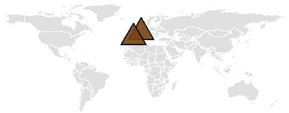

Pipeline 1 -

DWI artifact removal (step1)

- Denoising (optional): NLMEANS algorithm from the dipy project, with automatic estimation of noise variance using a coarse mask of white-matter (WM).

- Susceptibility-derived distortions: we applied the tool FUGUE from FSL. The fieldmap is computed using an in-house phase-unwrapping algorithm over the supplied field- map.

- Head motion: we used a dwi-to-dwi rigid-registration algorithm using ANTs. Settings for the pipeline, and the methodology used to accordingly rotate the b-vectors is contributed to the nipype tool, and actual code found in: https://github.com/oesteban/nipype/tree/enh/ReorganizeWorkflows.

Segmentation (step2)

- Preliminary image registration: the image provided in the challenge dataset was linearly registered to the artifact free b0 image using FSL

- White-matter mask registration: an in-house method for simultaneous segmentation and registration of dMRI images, is used to refine the fitting of three surfaces onto the dMRI data. The three surfaces are the boundaries of the ventricular system, the white-matter/gray-matter interface and the pial surface. Tissue fractions were then used to compute GM and WM labels in diffusion space.

Parcellation (step3)

- The MNI template was non-linearly registered to the undistorted B0 image using FSL's FNIRT tool. The transformation was then applied to the Harvard-Oxford cortical and subcortical atlases as well as the cerebellar label of the MNI structural atlas provided in the FSL toolbox. GM voxels were then labeled according to the closest values in these atlases, and assigning different labels for left and right hemispheres. Missing labels were

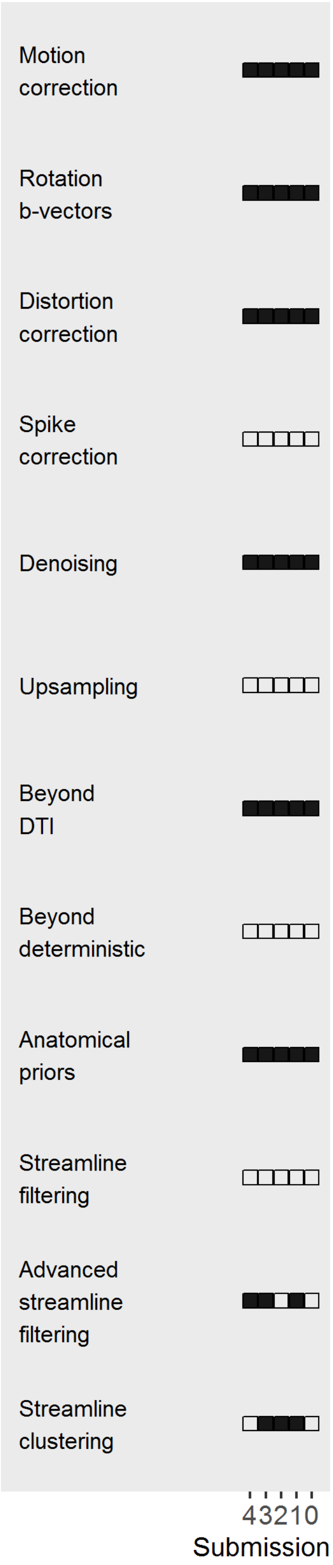

removed, leading to a subject atlas with 112 regions.

Reconstruction (step4)

- The reconstruction was implemented using a formulation that solves the fibre configuration of all voxels of interest simultaneously and imposes spatial regularisation directly on the fibre space. This reconstruction method allows us to exploit information from the neighbouring voxels, translating the natural smoothness of the anatomical fibre tracts through the brain into a certain *spatial coherence* of the FOD (Fiber Orientation Distribution function) in neighbouring voxels[42].

Tractography (step5)

For all methods below, tractography was restrained to the WM mask computed during the Preprocessing step.

. Gibbs : Gibbs global tractography was computed using MITK and the undistorted diffusion signal. The following parameters were used : particles had a length of 3mm, width of 1mm and a weight of 0.0008, start and ending temperatures were set to 0.1 and 0.001 respectively, the balance between external and internal energy was set to 0, the minimum fiber length to 20mm, the curvature threshold to 60° and the random seeds to 100. Fibres were then extended by half a voxel on each end, and we extracted the fibres that started and ended inside the GM mask.

. Streamlines : streamline tractography was performed using the fibre peaks generated in the reconstruction step. Tractography was done using the FACT algorithm implemented in MRTRix in order to generate 100’000 fibres. Fibres were then extended by half a voxel in both ends, and we extracted the fibres that started and ended inside the GM.

. Probabilistic tractography : probabilistic tractography was computed on the Spherical Harmonics representation of the ODF from the Reconstruction step. We used the iFOD2 algorithm implemented in MRTrix to generate 100’000 fibres. We then extended the fibres by half a voxel on both ends, and extracted a set of fibres that started and ended inside the GM mas

Superset (step6) – 16_0

- The three tractograms above were concatenated into one single tractogram called Superset1.trk

Pipeline 2 – 16_2

Steps 1 to 5 were followed as in pipeline 1, and the output of probabilistic tractography was clustered using Nibabel's QuickBundle tool, with a distance threshold of 4mm, before concatenating

the tractograms into a single superset. The trackfile was named Superset3.trk

Pipeline 3 – 16_4

Fibres in Superset1 were asigned a weight according to their contribution to the undistorted diffusion signal using COMMIT with default parameters[43]. The fibres were then replicated proportionaly to their assigned weight in a file called Superset1_COMMIT.trk

Pipeline 4 – 16_1

Steps 1 to 5 were followed as in Pipeline 1. Output tractograms from probabilistic and streamline tractography were clustered using QuickBundles, and the clustered tractograms concatenated with Gibb's output. Fibres in the resulting superset were asigned a weight according to their contribution to the undistorted diffusion signal using COMMIT with default parameters. The fibres were then replicated proportionaly to their assigned weight in a file called Superset2_COMMIT.trk

Pipeline 5 – 16_3

Fibres in Superset3 were asigned a weight according to their contribution to the undistorted diffusion signal using COMMIT with default parameters. The fibres were then replicated proportionaly to their assigned weight in a file called Superset3_COMMIT.trk

Team 17 – Submissions 17_0 to 17_4 (5 submissions)

M Barakovic, O Esteban, A Auria, A Lemkaddem, J-P Thiran

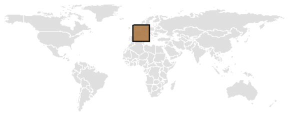

DWI artifact removal (step1)

- Denoising (optional): NLMEANS algorithm from the dipy project, with automatic estimation of noise variance using a coarse mask of white-matter (WM).

- Susceptibility-derived distortions: we applied the tool FUGUE from FSL. The fieldmap is computed using an in-house phase-unwrapping algorithm over the supplied field- map.

- Head motion: we used a dwi-to-dwi rigid-registration algorithm using ANTs. Settings for the pipeline, and the methodology used to accordingly rotate the b-vectors is contributed to the nipype tool, and actual code found in: https://github.com/oesteban/nipype/tree/enh/ReorganizeWorkflows.

Segmentation (step2)

- Preliminary image registration: the image provided in the challenge dataset was linearly registered to the artifact free b0 image using FSL

- White-matter mask registration: an in-house method for simultaneous segmentation and registration of dMRI images, is used to refine the fitting of three surfaces onto the dMRI data. The three surfaces are the boundaries of the ventricular system, the white-matter/gray-matter interface and the pial surface. Tissue fractions were then used to compute GM and WM labels in diffusion space.

Parcellation (step3)

- The MNI template was non-linearly registered to the undistorted B0 image using FSL's FNIRT tool. The transformation was then applied to the Harvard-Oxford cortical and subcortical atlases as well as the cerebellar label of the MNI structural atlas provided in the FSL toolbox. GM voxels were then labeled according to the closest values in these atlases, and assigning different labels for left and right hemispheres. Missing labels were removed, leading to a subject atlas with 112 regions.

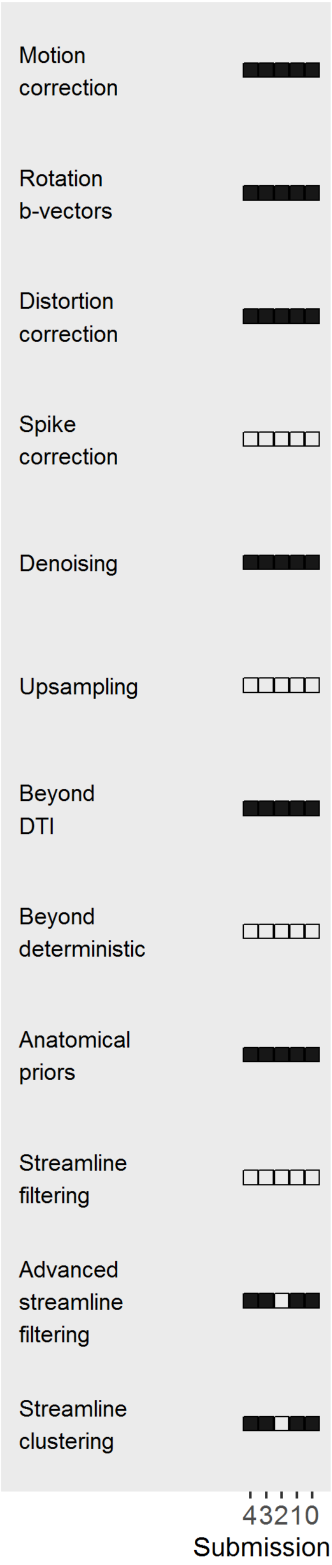

Reconstruction (step4)

- The reconstruction was implemented using a formulation that solves the fibre configuration of all voxels of interest simultaneously and imposes spatial regularization directly on the fibre space. This reconstruction method allows us to exploit information from the neighboring voxels, translating the natural smoothness of the anatomical fibre tracts through the brain into a certain *spatial coherence* of the FOD (Fiber Orientation Distribution function) in neighboring voxels[42].

Tractography (step5)

For all methods below, tractography was restrained to the WM mask computed during the Preprocessing step.

- Gibbs : Gibbs global tractography was computed using MITK and the undistorted diffusion signal. The following parameters were used : particles had a length of 3mm, width of 1mm and a weight of 0.0008, start and ending temperatures were set to 0.1 and 0.001 respectively, the balance between external and internal energy was set to 0, the minimum fiber length to 20mm, the curvature threshold to 60° and the random seeds to 100. Fibres were then extended by half a voxel on each end, and we extracted the fibres that started and ended inside the GM mask.

- Streamlines : streamline tractography was performed using the fibre peaks generated in the reconstruction step. Tractography was done using the FACT algorithm implemented in MRTRix in order to generate 100’000 fibres. Fibres were then extended by half a voxel in both ends, and we extracted the fibres that started and ended inside the GM.

- Probabilistic tractography : probabilistic tractography was computed on the Spherical Harmonics representation of the ODF from the Reconstruction step. We used the iFOD2 algorithm implemented in MRTrix to generate 100’000 fibres. We then extended the fibres by half a voxel on both ends, and extracted a set of fibres that started and ended inside the GM mask.

Superset (step6) – 17_2

- The three tractograms above were concatenated after clustering the probabilistic tractography and streamline outputs using Dipy’s QuickBundle tool, with a distance threshold of 4mm. The concatenated tractogram was saved as Superset2.trk

Pipeline 2 – 17_0

Steps 1 to 5 were followed as in Pipeline 1. The tractography outputs were concatenated in a single file without clustering, and the corresponding fibres were assigned a weight according to their contribution to the undistorted diffusion signal using a Monte-Carlo simulation with 0.4 probability of moving a fibre, 0.4 probability of changing the weight of a fibre, 0.15 probability to add a fibre and 0.05 probability of removing a fibre. We computed 5'000'000 iterations with an initial weight of

0.0071 for all fibres. The output fibres were then replicated proportionaly to their assigned weight in a file called Superset1_MCMC.trk

Pipeline 3 – 17_4

Fibres in Superset2 were asigned a weight according to their contribution to the undistorted diffusion signal using the same method as for Pipeline 7, with an initial weight of 0.0265 and 10'000'000 iterations. The fibres were then replicated proportionaly to their assigned weight in a file called Superset2_MCMC.trk

Pipeline 4 – 17_3

Steps 1 to 5 were followed as in Pipeline 1. The output of probabilistic tractography was clustered using QuickBundles and then concatenated with the streamline and Gibb's outputs. The fibres in the resulting tractogram were assigned a weight according to their contribution to the undistorted diffusion signal using the same method as for Pipeline 7 with an initial weight of 0.015 and 10'000'000 iterations. The fibres were then replicated proportionally to their assigned weight in a file called Superset3_MCMC.trk

Pipeline 5 – 17_1

Fibres in Superset2 were asigned a weight according to their contribution to the undistorted diffusion signal using a Monte-Carlo simulation with equal probability of 0.25 for all events and 10'000'000 iterations[44]. The fibres were then replicated proportionaly to their assigned weight in a file called Superset2_MCMC_0.25.trk

Team 18 – Submissions 18_0 to 18_4 (5 submissions)

H. E Cetingul, B Odry, B Mailhe, M S. Nadar

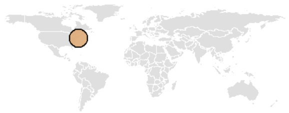

Pipeline description

STEP 1

- Two b0 images acquired in PE and reverse PE directions, respectively, are used to estimate a field map (FM) in rad/s and FM correction is applied to the DWI data. (FSL[35] TOPUP and FUGUE)
- Eddy current distortion correction is applied to the DWI data. (FSL[35] EDDY_CORRECT)
- A medical image denoising method developed in house is applied to the corrected DWI data.
- A brain mask is obtained from the corrected, denoised b0 image. (FSL[35] BET)
- T1w image is nonlinearly registered (via FSL[35] FLIRT and FNIRT) to the corrected b0 image (referred to as subject space).
- In house tissue segmentation (WM/GM/CSF) is applied to the warped T1w image to obtain WM and GM masks, as well as a WM/GM interface image.

STEP 2

- DT fitting is performed to processed DWI data to obtain a representative FA image. (FSL[35] DTIFIT)
- FA image of the JHU-DTI-Prob[45] atlas is nonlinearly registered to the FA image of the subject. (ANTS[46])
- 47 group-averaged WM tract maps in the atlas are warped to the subject space.

STEP 3

- MNI atlas is nonlinearly registered to the (warped) T1w image. (FSL FLIRT and FNIRT)
- AAL[47] atlas (in the MNI space by construction) is warped to the subject space.
- 47 group-averaged WM tracts in the JHU atlas are used to identify the AAL ROIs each tract is passing through.

STEP 4

- Processed DWI data, WM mask, WM/GM interface image, warped AAL ROIs and WM tract maps are used in the computation and filtering of the tractogram(s) via MRtrix[48].
    - Tractogram1 – 18_3: DT-based FACT with RK4 integration, WM mask as seed image and mask image, filtering using WM tract maps and AAL ROIs.
    - Tractogram2 – 18_1: CSD-based probabilistic tractography using iFOD1, WM/GM interface for seeding, WM mask for masking, filtering using WM tract maps and AAL ROIs.
    - Tractogram3– 18_0: CSD-based probabilistic

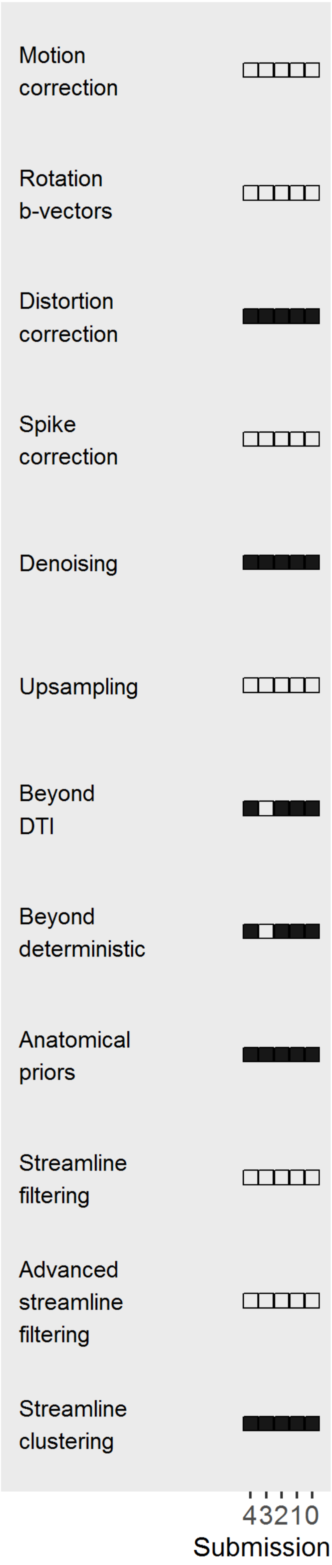

tractography using iFOD1, WM mask as seed image and mask image, filtering using WM tract maps and AAL ROIs.

- Tractogram4 – 18_4: CSD-based probabilistic tractography using iFOD2, WM/GM interface for seeding, WM mask for masking, filtering using WM tract maps and AAL ROIs.
- Tractogram5 – 18_2: CSD-based probabilistic tractography using iFOD2, WM mask as seed image and mask image, filtering using WM tract maps and AAL ROIs.

Team 19 – Submissions 19_0 to 19_2 (3 submissions)

F Pizzagalli, G Prasad, J Galvis, J Villalon

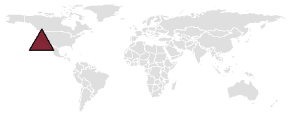

Pipeline 1: 19_1

1. Eddy Correction (FSL)[35]
2. Rotation of b-vectors using the transformation from the FSL eddy correction output.
3. EPI correction (FUGUE)[35]
    - This was done using an in-house EPI correction code named 3 Dimensional Mutal Information (DMI) by Alex Leow[49]
4. Hough Tractography[50,51]
    - Seeds: 29588
    - White matter mask (threshold>0.2)
        - Grey and white matter segmentation and intensity bias correction of T1 image using unified segmentation approach[52] as implemented in SPM12. White matter probability map was then binarized using a threshold of 0.2 and down-sampled to 2mm to create the white matter mask.
    - Hough tractography algorithm: briefly, this is an global probabilistic fiber tracking approach based on the Hough transform. This allows sorting the 3D curves in the volumes by computing a score from diffusion images. The curves with higher scores represent the potential anatomical connections.
    - Result: ISMRM_2015_challenge_Pipeline1_track.trk
5. Fiber Length Threshold: 18mm – 109mm
    - To remove small incorrect fibers from the periphery

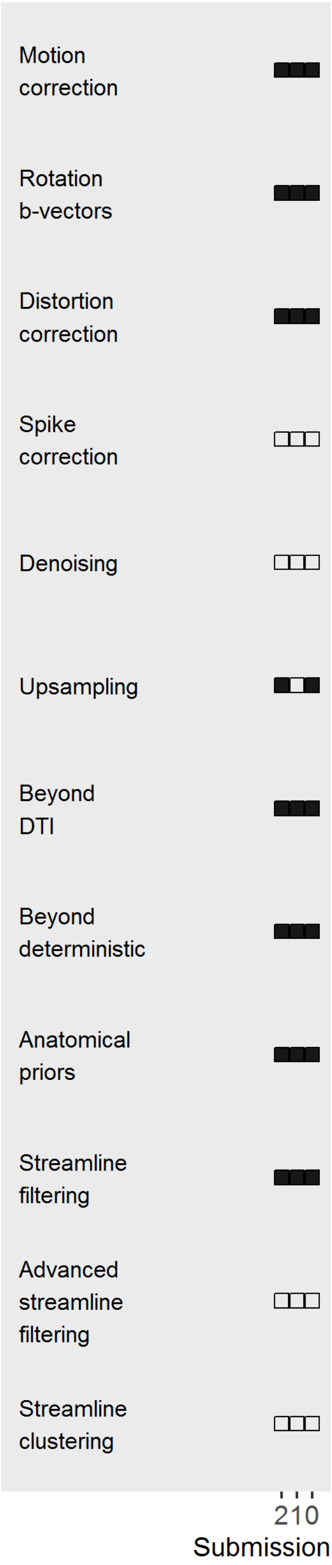

Pipeline 2: 19_2

1. Eddy Correction (FSL)[35]
2. Rotation of b-vectors using the transformation from the eddy correction tool.
3. EPI correction (FUGUE)[35]
4. Up-sampling to 1mm resolution using FSL's FLIRT.
5. Hough Tractography[50,51]
    - Seeds: 50000
    - GM+WM binary mask
        - Grey (GM) and white matter (WM) segmentation and intensity bias correction of T1 image using unified segmentation approach[52] as implemented in SPM12. Grey and white matter probability maps were then binarized using a threshold of 0.2 and down-sampled to 2mm. The mask was generated as GM + WM.
    - Result:

ISMRM_2015_challenge_Pipeline2_track.trk

5. Length Threshold: 18mm – 109mm

6. ROI filtering to also remove longer incorrect fibers from the outside of the brain

Pipeline 3: 19_0

1. Eddy Correction (FSL)[35]
2. Rotation of b-vectors using the transformation from the eddy correction tool.
3. EPI Correction (FUGUE)[35]
4. Registration to T1 and up-sampling to 1mm (flirt, normmi - 12dof)[53,54]
5. Hough Tractography[50,51]
   - Seeds: 150000
   - GM+WM binary mask
   - Result: ISMRM_2015_challenge_Pipeline3_track.trk
6. Length Threshold: 18mm – 109mm
7. ROI filtering to also remove longer incorrect fibers from the outside of the brain

Team 20 – Submissions 20_0 to 20_14 (15 submissions)

F De Santiago Requejo, P Luque Laguna, L Miguel Lacerda, R Barrett, F Dell' Acqua

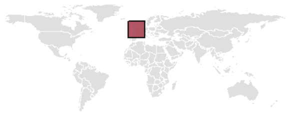

Pipelines description

Tract_file1 : DTI – 20_2

- Denoising was applied to diffusion data using Overcomplete Local PCA[3].
- TOPUP/EDDY correction from FSL and RESTORE from ExploreDTI[28] preprocessing were included (motion distortion, eddy_current, EPI geometrical distortions, spike correction).
- Data was processed using deterministic DTI tractography in ExploreDTI, with angle threshold of 45 degrees and FA stopping threshold of 0.15
- Semi-automatic pruning using manually selected regions of interest was applied to all datasets to filter out spurious streamlines and identify major white matter bundles.
- The data was processed using different combinations of preprocessing from FSL and ExploreDTI as described above to identify and the best reconstruction for each anatomical tract.

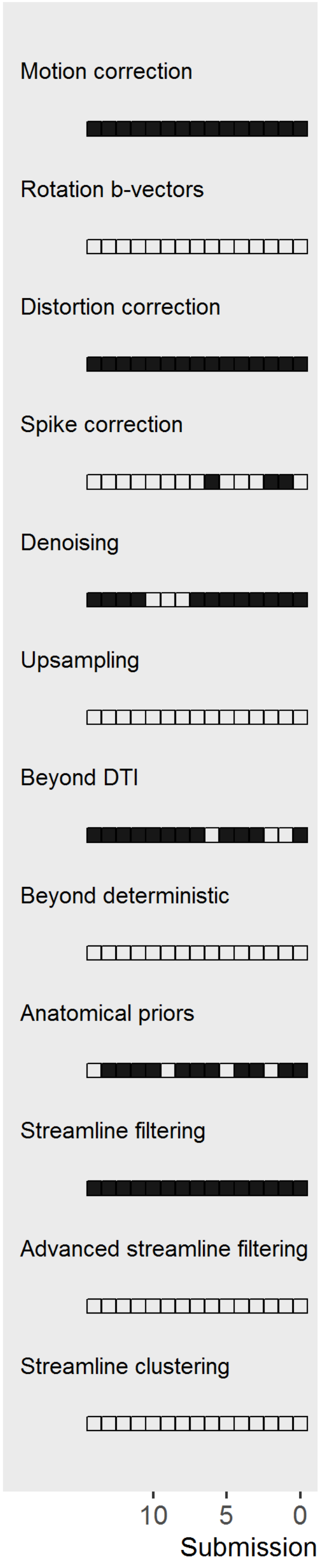

Tract_file2 : DTI_clean – 20_1

- Denoising was applied to diffusion data using Overcomplete Local PCA[3].
- TOPUP/EDDY correction from FSL and RESTORE from ExploreDTI[28] preprocessing were included (motion distortion, eddy_current, EPI geometrical distortions, spike correction).
- Data was processed using deterministic DTI tractography in ExploreDTI, with angle threshold of 45 degrees and FA stopping threshold of 0.15
- Semi-automatic pruning using manually selected regions of interest was applied to all datasets to filter out spurious streamlines and identify major white matter bundles.
- The data was processed using different combinations of preprocessing from FSL and ExploreDTI as described above to identify and the best reconstruction for each anatomical tract.
- An additional automatic filter was applied to remove spurious streamlines stopping and starting in either white matter regions, or starting and stopping in CSF.

Tract_file3 : DTI_cleanest – 20_6

- Denoising was applied to diffusion data using Overcomplete Local PCA[3].
- TOPUP/EDDY correction from FSL and RESTORE from ExploreDTI[28] preprocessing were included (motion

distortion, eddy_current, EPI geometrical distortions, spike correction).
- Data was processed using deterministic DTI tractography in ExploreDTI, with angle threshold of 45 degrees and FA stopping threshold of 0.15
- Semi-automatic pruning using manually selected regions of interest was applied to all datasets to filter out spurious streamlines and identify major white matter bundles.
- The data was processed using different combinations of preprocessing from FSL and ExploreDTI as described above to identify and the best reconstruction for each anatomical tract.
- An additional automatic filter was applied to remove spurious streamlines stopping in deep white matter regions, or starting and stopping in CSF.

Tract_file1 : baseline – 20_9

- TOPUP/EDDY correction from FSL follewed by standard ExploreDTI[28] preprocessing including (motion distortion, eddy_current and EPI geometrical distortions)
- Data was processed using Spherical Deconvolution with a damped Richardson-Lucy algorithm was applied to the data as described in Dell'Acqua et al.[55] using fibre-response model alpha = 2.5 and number of iterations = 600 to optimise angle resolution and noise stability.
- Tractography was performed as described in Dell'Acqua et al.[56], with absolute FOD threshold of 0.05 and angle threshold of 45degrees
- Semi-automatic pruning using manually selected regions of interest was applied to all datasets to filter out spurious streamlines and identify major white matter bundles.

Tract_file2 : LPCA_conservative – 20_5

- Denoising was applied to diffusion data using Overcomplete Local PCA[3].
- TOPUP/EDDY correction from FSL follewed by standard ExploreDTI[28] preprocessing including (motion distortion, eddy_current and EPI geometrical distortions)
- Data was processed using Spherical Deconvolution with a damped Richardson-Lucy algorithm was applied to the data as described in Dell'Acqua et al.[55] using fibre-response model alpha = 2.5 and number of iterations = 700 to optimise angle resolution and noise stability.
- Tractography was performed as described in Dell'Acqua et al.[56], with absolute FOD threshold of 0.05 and angle threshold of 45degrees
- Semi-automatic pruning using manually selected regions of interest was applied to all datasets to filter out spurious streamlines and identify major white matter bundles.

Tract_file3 : LPCA_risky – 20_14

- Denoising was applied to diffusion data using Overcomplete Local PCA[3].
- TOPUP/EDDY correction from FSL follewed by standard ExploreDTI preprocessing including (motion distortion, eddy_current and EPI geometrical distortions)
- Data was processed using Spherical Deconvolution with a damped Richardson-Lucy algorithm was applied to the data as described in Dell'Acqua et al.[55] using fibre-response model alpha = 2.5 and number of iterations = 800 to optimise angle resolution and noise stability.
- Tractography was performed as described in Dell'Acqua et al.[56], with absolute FOD threshold of 0.05 and angle threshold of 45degrees
- Semi-automatic pruning using manually selected regions of interest was applied to all datasets to filter out spurious streamlines and identify major white matter bundles.

Tract_file4 : Chimera – 20_0

- Denoising was applied to diffusion data using Overcomplete Local PCA[3].
- TOPUP/EDDY correction from FSL followed by standard ExploreDTI preprocessing including (motion distortion, eddy_current and EPI geometrical distortions)
- Data was processed using Spherical Deconvolution with a damped Richardson-Lucy algorithm was applied to the data as described in Dell'Acqua et al.[55] using fibre-response model alpha = 2.5 and number of iterations ranging from 600 - 800 to optimise angle resolution and noise stability.
- Tractography was performed as described in Dell'Acqua et al.[56], with absolute FOD threshold of 0.05 and angle threshold of 45degrees
- Semi-automatic pruning using manually selected regions of interest was applied to all datasets to filter out spurious streamlines and identify major white matter bundles.
- The data was processed with and without denoising, using both conservative and liberal spherical deconvolution settings to identify and the best reconstruction for each anatomical tract.

Tract_file1 : baseline_clean – 20_10

- TOPUP/EDDY correction from FSL followed by standard ExploreDTI[28] preprocessing including (motion distortion, eddy_current and EPI geometrical distortions)
- Data was processed using Spherical Deconvolution with a damped Richardson-Lucy algorithm was applied to the data as described in Dell'Acqua et al.[55] using fibre-response model alpha = 2.5 and number of iterations = 600 to optimise angle resolution and noise stability.
- Tractography was performed as described in Dell'Acqua et al.[56], with absolute FOD threshold of 0.05 and angle threshold of 45degrees
- Semi-automatic pruning using manually selected regions of interest was applied to all datasets to filter out spurious streamlines and identify major white matter bundles.
- An additional automatic filter was applied to remove spurious streamlines starting and stopping in either deep white matter regions or CSF.

Tract_file2 : LPCA_conservative_clean – 20_4

- Denoising was applied to diffusion data using Overcomplete Local PCA[3].
- TOPUP/EDDY correction from FSL followed by standard ExploreDTI[28] preprocessing including (motion distortion, eddy_current and EPI geometrical distortions)
- Data was processed using Spherical Deconvolution with a damped Richardson-Lucy algorithm was applied to the data as described in Dell'Acqua et al.[55] using fibre-response model alpha = 2.5 and number of iterations = 700 to optimise angle resolution and noise stability.
- Tractography was performed as described in Dell'Acqua et al.[56], with absolute FOD threshold of 0.05 and angle threshold of 45degrees
- Semi-automatic pruning using manually selected regions of interest was applied to all datasets to filter out spurious streamlines and identify major white matter bundles.
- An additional automatic filter was applied to remove spurious streamlines starting and stopping in either deep white matter regions or CSF.

Tract_file3 : LPCA_risky_clean – 20_3

- Denoising was applied to diffusion data using Overcomplete Local PCA[3].
- TOPUP/EDDY correction from FSL followed by standard ExploreDTI preprocessing including (motion distortion, eddy_current and EPI geometrical distortions)
- Data was processed using Spherical Deconvolution with a damped Richardson-Lucy algorithm was applied to the data as described in Dell'Acqua et al.[55] using fibre-response model alpha = 2.5 and number of iterations = 800 to optimise angle resolution and noise stability.

- Tractography was performed as described in Dell'Acqua et al.[56], with absolute FOD threshold of 0.05 and angle threshold of 45degrees
- Semi-automatic pruning using manually selected regions of interest was applied to all datasets to filter out spurious streamlines and identify major white matter bundles.
- An additional automatic filter was applied to remove spurious streamlines starting and stopping in either deep white matter regions or CSF.

Tract_file4 : Chimera_clean – 20_13

- Denoising was applied to diffusion data using Overcomplete Local PCA[3].
- TOPUP/EDDY correction from FSL follewed by standard ExploreDTI preprocessing including (motion distortion, eddy_current and EPI geometrical distortions)
- Data was processed using Spherical Deconvolution with a damped Richardson-Lucy algorithm was applied to the data as described in Dell'Acqua et al.[55] using fibre-response model alpha = 2.5 and number of iterations ranging from 600 - 800 to optimise angle resolution and noise stability.
- Tractography was performed as described in Dell'Acqua et al.[56], with absolute FOD threshold of 0.05 and angle threshold of 45degrees
- Semi-automatic pruning using manually selected regions of interest was applied to all datasets to filter out spurious streamlines and identify major white matter bundles.
- The data was processed with and without denoising, using both conservative and liberal spherical deconvolution settings to identify and the best reconstruction for each anatomical tract.
- An additional automatic filter was applied to remove spurious streamlines starting and stopping in either deep white matter regions or CSF.

Tract_file1 : baseline_cleanest – 20_8

- TOPUP/EDDY correction from FSL follewed by standard ExploreDTI[28] preprocessing including (motion distortion, eddy_current and EPI geometrical distortions)
- Data was processed using Spherical Deconvolution with a damped Richardson-Lucy algorithm was applied to the data as described in Dell'Acqua et al.[55] using fibre-response model alpha = 2.5 and number of iterations = 600 to optimise angle resolution and noise stability.
- Tractography was performed as described in Dell'Acqua et al.[56], with absolute FOD threshold of 0.05 and angle threshold of 45degrees
- Semi-automatic pruning using manually selected regions of interest was applied to all datasets to filter out spurious streamlines and identify major white matter bundles.
- An additional automatic filter was applied to remove spurious streamlines stopping in deep white matter regions, or starting and stopping in CSF.

Tract_file2 : LPCA_conservative_cleanest – 20_7

- Denoising was applied to diffusion data using Overcomplete Local PCA[3].
- TOPUP/EDDY correction from FSL follewed by standard ExploreDTI[28] preprocessing including (motion distortion, eddy_current and EPI geometrical distortions)
- Data was processed using Spherical Deconvolution with a damped Richardson-Lucy algorithm was applied to the data as described in Dell'Acqua et al.[55] using fibre-response model alpha = 2.5 and number of iterations = 700 to optimise angle resolution and noise stability.

- Tractography was performed as described in Dell'Acqua et al.[56], with absolute FOD threshold of 0.05 and angle threshold of 45degrees
- Semi-automatic pruning using manually selected regions of interest was applied to all datasets to filter out spurious streamlines and identify major white matter bundles.
- An additional automatic filter was applied to remove spurious streamlines stopping in deep white matter regions, or starting and stopping in CSF.

Tract_file3 : LPCA_risky_cleanest – 20_11

- Denoising was applied to diffusion data using Overcomplete Local PCA[3].
- TOPUP/EDDY correction from FSL follewed by standard ExploreDTI preprocessing including (motion distortion, eddy_current and EPI geometrical distortions)
- Data was processed using Spherical Deconvolution with a damped Richardson-Lucy algorithm was applied to the data as described in Dell'Acqua et al.[55] using fibre-response model alpha = 2.5 and number of iterations = 800 to optimise angle resolution and noise stability.
- Tractography was performed as described in Dell'Acqua et al.[56], with absolute FOD threshold of 0.05 and angle threshold of 45degrees
- Semi-automatic pruning using manually selected regions of interest was applied to all datasets to filter out spurious streamlines and identify major white matter bundles.
- An additional automatic filter was applied to remove spurious streamlines stopping in deep white matter regions, or starting and stopping in CSF.

Tract_file4 : Chimera_cleanest – 20_12

- Denoising was applied to diffusion data using Overcomplete Local PCA[3].
- TOPUP/EDDY correction from FSL follewed by standard ExploreDTI preprocessing including (motion distortion, eddy_current and EPI geometrical distortions)
- Data was processed using Spherical Deconvolution with a damped Richardson-Lucy algorithm was applied to the data as described in Dell'Acqua et al.[55] using fibre-response model alpha = 2.5 and number of iterations ranging from 600 - 800 to optimise angle resolution and noise stability.
- Tractography was performed as described in Dell'Acqua et al.[56], with absolute FOD threshold of 0.05 and angle threshold of 45degrees
- Semi-automatic pruning using manually selected regions of interest was applied to all datasets to filter out spurious streamlines and identify major white matter bundles.
- The data was processed with and without denoising, using both conservative and liberal spherical deconvolution settings to identify and the best reconstruction for each anatomical tract.
- An additional automatic filter was applied to remove spurious streamlines stopping in deep white matter regions, or starting and stopping in CSF.

Supplementary Note 2: Extended tractography experiments

Tractography on ground truth directions

We ran additional tractography experiments directly on the *ground truth field of fiber orientations*. Those orientations were found using the ground-truth bundles, by extracting the main directions of bundles going through each voxel. The experiments were performed using multiple resoltuions of the ground truth vector field (2mm, 1.75mm, 1.5mm, 1.25mm, 1.0mm, 0.75mm and 0.5mm) and two independent implementations of deterministic streamline tractography methods (GT_1 and GT_2). GT_1 is an in-house development of the Sherbrooke Connectivity Imaging Lab. GT_2 is implemented and openly available in MITK (mitk.org). Both tractography algorithms used the orientations as input. The two methods used the following parameters for all datasets:

GT1:

- 100k streamlines seeded in the white matter
- Step size 0.2mm
- Max angle between 2 steps: 45 degrees
- Min/max lenght of streamlines: 10mm/300mm

GT2:

- 100k streamlines seeded in the white matter
- Step size 0.5 * voxel size
- Max angle between 2 steps: 45 degrees
- Min/max lenght of kept streamlines: 20mm to 200mm

The results of these experiments can be found in Supplementary Figure 5.

Anatomically constrained and global tractography

We ran additional tractography experiments for an in-depth analysis of the benefits related to global tractography as well as the application of anatomical constraints during tractography. The experiments were performed using the following methods:

1. An openly available approach for anatomically constrained tractography (ACT) that is based on deterministic ("ACT Deterministic") or probabilistic ("ACT Probabilistic") streamline tractography.[57]
2. An MITK-based approach that implements global tractography[58] ("MITK Global")
3. An MRtrix-based approach that implements global tractography[59] ("MRtrix Global")

We newly implemented two tractography approaches that combine the above cited global tractography methods with posteriorly applied anatomical constraints. This was achieved on basis of the constraints described by Smith et al.[57]

4. "MITK Global" with anatomical constraints ("MITK Global ACT")
5. "MRtrix Global" with anatomical constraints ("MRtrix Global ACT")

Experiments were performed following these steps: First, the diffusion-weighted image was denoised and corrected for headmotion and distortions using MRtrix (dwidenoise & dwipreproc, http://www.mrtrix.org/). Multi-tissue response functions were estimated using MRtrix (dwi2response[60]). Single shell spherical deconvolution was performed using the white matter

response function. A map of white matter, gray matter and CSF was estimated from the T1 image using the MRtrix command 5ttgen. Deterministic and probabilistic (iFOD2) ACT[57] was performed using MRtrix with 100000 seeds randomly placed inside the brain mask. A minimum fiber length threshold of 20mm was applied. MRtrix global tractography[59] was performed using a 2-tissue WM/CSF-model within a WM mask, as recommended by the MRtrix team. The white matter mask was estimated from the T1 image. The particle weight was set to 0.05 and the number of iterations to 1e9. MITK global tractography[58] was performed on a Q-Ball reconstruction of the diffusion-weighted image within the whole brain. The particle weight was set to 0.0007, the particle width to 0.5 and the number of iterations to 1e9.

The results of these experiments can be found in Supplementary Figure 6.

Supplementary Note 3: Statistical analysis of processing steps

Effects of the methodological setup of the different submissions on the results were investigated in a multivariable linear mixed model (Table 1). While spike correction and denoising techniques significantly increased the volumetric overreach of identified bundles (mean effect: 10% ± 9% and 12% ± 9%, respectively), motion correction, rotation of b-vectors, distortion correction and upsampling had no significant effect on the outcome.

The diffusion modeling method had a striking effect on the overall scores. Pipelines that applied pure DTI modeling without locally resolving crossing fiber situations showed a better valid connection ratio (mean effect: 12% ± 13%), identified fewer invalid bundles (mean effect: -22 ± 35 bundles) and produced less bundle overreach (mean effect: -15% ± 9%) than other pipelines. More sophisticated models, on the other hand, improved the bundle overlap scores (mean effect: 13% ± 11%) and valid bundle counts (mean effect: 2.2 ± 2.6 bundles).

We find similar effects for probabilistic versus deterministic tractography methods. Deterministic algorithms produced significantly lower invalid connection counts (mean effect: -10% ± 13%), invalid bundle counts (mean effect: -41 ± 35 bundles), and bundle overreach (mean effect: -17% ± 9%) while reaching lower bundle overlap scores (mean effect: -12% ± 11%).

Tractogram post-processing also significantly influenced the results. Manual editing of tractograms following anatomical priors had a negative impact on the number of valid bundles identified (mean effect: -3.8 ± 2.6 bundles) and on the bundle overlap (mean effect: -15% ± 11%). However, such techniques showed a positive impact on the average bundle overreach (mean effect: -16% ± 9%). Basic streamline filtering techniques such as length thresholds on the streamlines had a positive effect on the percentage of valid connections (mean effect: 21% ± 13%). Advanced streamline filtering techniques such as *Spherical-deconvolution Informed Filtering of Tractograms* (SIFT) or *Convex Optimization Modeling for Microstructure Informed Tractography* (COMMIT) significantly improved the valid bundle count (mean effect: 2.8 ± 2.7 bundles), while negatively impacting the valid connection ratio (mean effect: -11% ± 13%). Clustering of streamlines had no significant effect on the outcome.

Supplementary Note 4: Description of relaxed scoring

Valid connection (VC) and valid bundle (VB) scoring:

To overcome the limits of the classical Tractometer metrics, we use a relaxed streamlines assignment technique. This technique is based on a bundle recognition technique[61]. Each streamline in a submission is compared to the 25 ground-truth bundles. The streamline is assigned to its closest ground-truth bundle, if it is within a specific, bundle-specific distance threshold. If the streamline does not match closely enough with any GT bundle, it will be set aside for further classification in the invalid bundle (IB) scoring phase.

Invalid bundle (IB) scoring:

To overcome the same limitations as for the VC assignment phase, we implement an invalid bundle assignment technique. All streamlines that have been set aside during the VC assignment phase are now processed as a whole. Candidate streamlines shorter than 35mm are discarded following a heuristic based on the length distribution of ground-truth streamlines. A QuickBundles-based clustering algorithm is then applied to all remaining candidates using a threshold of 20mm, to cluster all of them in similarly shaped and positioned candidate invalid bundles. Candidate invalid bundles containing only one streamline are discarded, since, from observation, an IB with only 1 streamline is most likely an outlier.

To assign a meaningful and representative ID to each candidate IB, we create the set of ending regions of all ground-truth VBs. In this set, there are 2 regions for each ground-truth bundle, representing the « head » and « tail » of a bundle. For a specific IB candidate, for each end of each streamline, we find the ending region that is closest to that end. The IB is then named after the pair representing the most found region, for both ends of the bundle. If multiple candidates are assigned to the same pair of regions, they are merged.

Finally, streamlines that are discarded based on their length or due to the fact that they create singleton IBs are assigned to the NC category.

Supplementary Notes 5: Selection of best-performing submissions

There are clear trade-offs that have to made between optimizing different metrics. A low percentage of invalid connections, for example, is hard to achieve if the valid connections are expected to cover large parts of the bundle volumes. A tractography pipeline good for the first is rarely optimal for the latter. Here we provide an overview of submissions that achieved extraordinarily good results in certain categories.

> ***i) Most number of valid bundles***: 24/25 valid bundles. 10 pipelines (contributed by 3 teams) achieved this result. Since all those pipelines used similar approaches, we chose one to provide this picture of results (submission 0 from team 9, HARDI deterministic tractography). We used this specific one since it was the one with most streamlines, showing a trade-off between VC and number of streamlines. This pipeline used state-of-the-art pre-processing (motion correction, rotated b-vectors, distortion correction, denoising and upsampling to 1x1x1 space), fiber ODFs were reconstructed, 2 million streamlines were generated from a white matter seeding strategy and deterministic tractography. The final tractogram was post-processed with a QuickBundles clustering algorithm with distance 2.5 mm to clean up some of the spurious streamlines. 52% of the submitted streamlines were valid with 50% overlap (bundle coverage). 41% of the connections were invalid accounting for 127 invalid bundles. Mean overreach was 62%. As seen in Figure 3 (third column), large extents of bundles are found (curving and fanning parts) and no streamline was found to reconstruct the posterior commissure.
>
> ***ii a) Most number of valid connections and consequently, least number of invalid by a HARDI pipeline:*** 92% valid connections / 8% invalid connections. The pipeline that achieved this used motion correction and upsampling to 1x1x1 space (submission 0 from team 3, HARDI deterministic tractography). The ODFs were reconstructed with generalized q-sampling and a white matter seeding was used with deterministic tractography. Only the 50 largest clusters were kept and small streamline count clusters were pruned out. This resulted in the best valid connection score of 92% and only 8% invalid connections. However, only 21 out of the 25 valid bundles were found with a mean overlap of 45%. In general, a high percentage of correct streamlines must be seen in context of the other metrics to ensure proper streamline distribution over the bundles. In case of this submission, the 10 smallest valid bundles found by the submission make up for 2% of the valid connections, while the 10 smallest existing bundles in the ground truth make up for 14% of the existing connections. Even with 8% of invalid connections, 28 invalid bundles were found (second best value after submission 4 of the same team with 27 invalid bundles). Mean overreach was 60%.
>
> ***ii b) Most number of valid connections and consequently, least number of invalid by a DTI pipeline:*** 85% valid connections / 15% invalid connections. The pipeline that achieved this used motion correction, distortion correction, denoising and a DTI reconstruction (submission 6 from team 20, DTI deterministic tractography). Deterministic tractography was followed by a careful manual editing and semi-automatic virtual dissection to clean up the submitted tractogram. This resulted in only 15% invalid connections with 37 invalid bundles and a mean bundle overreach of 12%. 23 out of the 25 valid bundles were recovered with 85% valid connections and a mean bundle overlap of 29%. Hence, very similar scores as the HARDI version of ii) above, but here, with DTI and dedicated post-processing clean up. As seen in Figure 3 second column, although many bundles are found, they are limited in their spatial extent, pushed away from crossing areas and limited in the fanning areas. However, most straight parts of the bundles are recovered and well filled.

iii) Most overlap with ground truth: 77%. The pipeline that achieved this used a spike detection/correction strategy, motion/distortion correction, denoising and a fiber ODF reconstruction in 1x1x1 space (submission 2 of team 12, HARDI probabilistic tractography). A probabilistic tractography algorithm was used with white matter seeding strategy and post-processing cleanup with anatomical priors. A mean 77% bundle coverage overlap was reached with 23/25 valid bundles and 45% valid connections. However, 44% invalid connections and 198 invalid bundles were reconstructed with a mean bundle overreach of 89%. Figure 3 last column shows reconstructed bundles of this submission.

Supplementary References

1. Houde, J.-C. *et al.* Tractography Challenge ISMRM 2015 Code of the Scoring System v1.0.1. *Zenodo* (2017). doi:10.5281/zenodo.810130
2. Maier-Hein, K. H. *et al.* Tractography Challenge ISMRM 2015 Videos. *Zenodo* (2017). doi:10.5281/zenodo.580067
3. Manjón, J. V. *et al.* Diffusion Weighted Image Denoising Using Overcomplete Local PCA. *PLOS ONE* **8,** e73021 (2013).
4. Lam, F. *et al.* Denoising Diffusion-Weighted Magnitude MR Images using Rank and Edge Constraints. *Magn. Reson. Med.* **71,** 1272–1284 (2014).
5. Smith, S. M. *et al.* Advances in functional and structural MR image analysis and implementation as FSL. *NeuroImage* **23 Suppl 1,** S208-219 (2004).
6. Garyfallidis, E. *et al.* Dipy, a library for the analysis of diffusion MRI data. *Front. Neuroinformatics* **8,** 8 (2014).
7. Cox, R. W. AFNI: software for analysis and visualization of functional magnetic resonance neuroimages. *Comput. Biomed. Res. Int. J.* **29,** 162–173 (1996).
8. Tax, C. M. W., Jeurissen, B., Vos, S. B., Viergever, M. A. & Leemans, A. Recursive calibration of the fiber response function for spherical deconvolution of diffusion MRI data. *NeuroImage* **86,** 67–80 (2014).
9. Tournier, J.-D., Calamante, F. & Connelly, A. MRtrix: Diffusion tractography in crossing fiber regions. *Int. J. Imaging Syst. Technol.* **22,** 53–66 (2012).
10. Chamberland, M., Whittingstall, K., Fortin, D., Mathieu, D. & Descoteaux, M. Real-time multi-peak tractography for instantaneous connectivity display. *Front. Neuroinformatics* **8,** 59 (2014).
11. Yeh, F.-C., Wedeen, V. J. & Tseng, W.-Y. I. Generalized q-sampling imaging. *IEEE Trans. Med. Imaging* **29,** 1626–1635 (2010).
12. Yeh, F.-C. & Tseng, W.-Y. I. Sparse Solution of Fiber Orientation Distribution Function by Diffusion Decomposition. *PLOS ONE* **8,** e75747 (2013).

13. Yeh, F.-C., Verstynen, T. D., Wang, Y., Fernández-Miranda, J. C. & Tseng, W.-Y. I. Deterministic Diffusion Fiber Tracking Improved by Quantitative Anisotropy. *PLOS ONE* **8,** e80713 (2013).

14. Kent, B. P., Rinaldo, A., Yeh, F.-C. & Verstynen, T. Mapping Topographic Structure in White Matter Pathways with Level Set Trees. *PLOS ONE* **9,** e93344 (2014).

15. Behrens, T. E. J., Berg, H. J., Jbabdi, S., Rushworth, M. F. S. & Woolrich, M. W. Probabilistic diffusion tractography with multiple fibre orientations: What can we gain? *NeuroImage* **34,** 144–155 (2007).

16. Manjón, J. V., Coupé, P., Buades, A., Louis Collins, D. & Robles, M. New methods for MRI denoising based on sparseness and self-similarity. *Med. Image Anal.* **16,** 18–27 (2012).

17. Andersson, J. L. R., Skare, S. & Ashburner, J. How to correct susceptibility distortions in spin-echo echo-planar images: application to diffusion tensor imaging. *NeuroImage* **20,** 870–88 (2003).

18. Leemans, A. & Jones, D. K. The B-matrix must be rotated when correcting for subject motion in DTI data. *Magn. Reson. Med.* **61,** 1336–1349 (2009).

19. Hutton, C. *et al.* Image distortion correction in fMRI: A quantitative evaluation. *NeuroImage* **16,** 217–240 (2002).

20. Wu, Y., Feng, Y., Li, F. & Westin, C. F. Global consistency spatial model for fiber orientation distribution estimation. in *IEEE 12th International Symposium on Biomedical Imaging (ISBI)* 1180–1183 (2015).

21. St-Jean, S., Coupé, P. & Descoteaux, M. Non Local Spatial and Angular Matching: Enabling higher spatial resolution diffusion MRI datasets through adaptive denoising. *Med. Image Anal.* **32,** 115–130 (2016).

22. Deslauriers-Gauthier, S., Marziliano, P., Paquette, M. & Descoteaux, M. The application of a new sampling theorem for non-bandlimited signals on the sphere: Improving the recovery of crossing fibers for low b-value acquisitions. *Med. Image Anal.* **30,** 46–59 (2016).

23. Girard, G., Whittingstall, K., Deriche, R. & Descoteaux, M. Towards quantitative connectivity analysis: reducing tractography biases. *Neuroimage* **98,** 266–278 (2014).

24. Rheault, F., Houde, J.-C. & Descoteaux, M. Real time interaction with millions of streamlines. in *proc. of ISMRM* **23,** 6005 (2015).

25. Garyfallidis, E., Brett, M., Correia, M. M., Williams, G. B. & Nimmo-Smith, I. Quickbundles, a method for tractography simplification. *Front. Neurosci.* **6,** 175 (2012).

26. Aja-Fernández, S., Niethammer, M., Kubicki, M., Shenton, M. E. & Westin, C.-F. Restoration of DWI data using a Rician LMMSE estimator. *IEEE Trans. Med. Imaging* **27,** 1389–1403 (2008).

27. Froeling, M. *et al.* Diffusion-tensor MRI reveals the complex muscle architecture of the human forearm. *J. Magn. Reson. Imaging* **36,** 237–248 (2012).

28. Leemans, A., Jeurissen, B., Sijbers, J. & Jones, D. K. ExploreDTI: a graphical toolbox for processing, analyzing, and visualizing diffusion MR data. in *17th Annual Meeting of Intl Soc Mag Reson Med* **209,** 3537 (2009).

29. Rohde, G. K., Barnett, A. S., Basser, P. J., Marenco, S. & Pierpaoli, C. Comprehensive approach for correction of motion and distortion in diffusion-weighted MRI. *Magn. Reson. Med.* **51,** 103–114 (2004).

30. Irfanoglu, M. O., Walker, L., Sarlls, J., Marenco, S. & Pierpaoli, C. Effects of image distortions originating from susceptibility variations and concomitant fields on diffusion MRI tractography results. *Neuroimage* **61,** 275–288 (2012).

31. Tax, C. M. W., Otte, W. M., Viergever, M. A., Dijkhuizen, R. M. & Leemans, A. REKINDLE: Robust extraction of kurtosis INDices with linear estimation. *Magn. Reson. Med.* **73,** 794–808 (2015).

32. Veraart, J., Sijbers, J., Sunaert, S., Leemans, A. & Jeurissen, B. Weighted linear least squares estimation of diffusion MRI parameters: strengths, limitations, and pitfalls. *NeuroImage* **81,** 335–346 (2013).

33. Tournier, J.-D., Calamante, F. & Connelly, A. Robust determination of the fibre orientation distribution in diffusion MRI: non-negativity constrained super-resolved spherical deconvolution. *NeuroImage* **35,** 1459–1472 (2007).

34. Zhang, Y., Brady, M. & Smith, S. Segmentation of brain MR images through a hidden Markov random field model and the expectation-maximization algorithm. *IEEE Trans. Med. Imaging* **20,** 45–57 (2001).

35. Jenkinson, M., Beckmann, C. F., Behrens, T. E., Woolrich, M. W. & Smith, S. M. Fsl. *Neuroimage* **62,** 782–790 (2012).

36. Coupé, P. *et al.* An optimized blockwise nonlocal means denoising filter for 3-D magnetic resonance images. *Ieee Trans. Med. Imaging* **27,** 425–441 (2008).

37. Girard, G., Whittingstall, K., Deriche, R. & Descoteaux, M. Towards quantitative connectivity analysis: reducing tractography biases. *NeuroImage* **98,** 266–278 (2014).

38. Holland, D., Kuperman, J. M. & Dale, A. M. Efficient correction of inhomogeneous static magnetic field-induced distortion in Echo Planar Imaging. *Neuroimage* **50,** 175–183 (2010).

39. Smith, R. E., Tournier, J.-D., Calamante, F. & Connelly, A. Anatomically-constrained tractography: improved diffusion MRI streamlines tractography through effective use of anatomical information. *NeuroImage* **62,** 1924–1938 (2012).

40. Smith, R. E., Tournier, J.-D., Calamante, F. & Connelly, A. SIFT: spherical-deconvolution informed filtering of tractograms. *Neuroimage* **67,** 298–312 (2013).

41. Tournier, J. D., Calamante, F. & Connelly, A. Improved probabilistic streamlines tractography by 2nd order integration over fibre orientation distributions. in *Proc. 18th Annual Meeting of the Intl. Soc. Mag. Reson. Med.(ISMRM)* 1670 (2010).

42. Auría, A., Daducci, A., Thiran, J.-P. & Wiaux, Y. Structured sparsity for spatially coherent fibre orientation estimation in diffusion MRI. *NeuroImage* **115,** 245–255 (2015).

43. Daducci, A., Dal Palù, A., Lemkaddem, A. & Thiran, J.-P. COMMIT: convex optimization modeling for microstructure informed tractography. *IEEE Trans. Med. Imaging* **34,** 246–257 (2015).

44. Lemkaddem, A., Skiöldebrand, D., Dal Palú, A., Thiran, J.-P. & Daducci, A. Global tractography with embedded anatomical priors for quantitative connectivity analysis. *Front. Neurol.* **5,** 232 (2014).

45. Zhang, Y. *et al.* Atlas-guided tract reconstruction for automated and comprehensive examination of the white matter anatomy. *Neuroimage* **52,** 1289–1301 (2010).

46. Avants, B. B., Epstein, C. L., Grossman, M. & Gee, J. C. Symmetric diffeomorphic image registration with cross-correlation: evaluating automated labeling of elderly and neurodegenerative brain. *Med. Image Anal.* **12,** 26–41 (2008).

47. Tzourio-Mazoyer, N. *et al.* Automated anatomical labeling of activations in SPM using a macroscopic anatomical parcellation of the MNI MRI single-subject brain. *Neuroimage* **15,** 273–289 (2002).

48. Tournier, J.-D., Calamante, F. & Connelly, A. MRtrix: Diffusion tractography in crossing fiber regions. *Int. J. Imaging Syst. Technol.* **22,** 53–66 (2012).

49. Leow, A. D. *et al.* Statistical properties of Jacobian maps and the realization of unbiased large-deformation nonlinear image registration. *IEEE Trans. Med. Imaging* **26,** 822–832 (2007).

50. Aganj, I. *et al.* A Hough transform global probabilistic approach to multiple-subject diffusion MRI tractography. *Med. Image Anal.* **15,** 414–425 (2011).

51. Prasad, G., Nir, T. M., Toga, A. W. & Thompson, P. M. Tractography density and network measures in Alzheimer's disease. in *Biomedical Imaging (ISBI), 2013 IEEE 10th International Symposium on* 692–695 (IEEE, 2013).

52. Ashburner, J. & Friston, K. J. Unified segmentation. *Neuroimage* **26,** 839–851 (2005).

53. Jenkinson, M., Bannister, P., Brady, M. & Smith, S. Improved optimization for the robust and accurate linear registration and motion correction of brain images. *Neuroimage* **17,** 825–841 (2002).

54. Jenkinson, M. & Smith, S. A global optimisation method for robust affine registration of brain images. *Med. Image Anal.* **5,** 143–156 (2001).

55. Dell'Acqua, F. *et al.* A modified damped Richardson–Lucy algorithm to reduce isotropic background effects in spherical deconvolution. *Neuroimage* **49,** 1446–1458 (2010).

56. Dell'Acqua, F., Simmons, A., Williams, S. C. & Catani, M. Can spherical deconvolution provide more information than fiber orientations? Hindrance modulated orientational anisotropy, a true-

tract specific index to characterize white matter diffusion. *Hum. Brain Mapp.* **34,** 2464–2483 (2013).

57. Smith, R. E., Tournier, J. D., Calamante, F. & Connelly, A. Anatomically-constrained tractography: improved diffusion MRI streamlines tractography through effective use of anatomical information. *Neuroimage* **62,** 1924–38 (2012).
58. Reisert, M. *et al.* Global fiber reconstruction becomes practical. *Neuroimage* **54,** 955–962 (2011).
59. Christiaens, D. *et al.* Global tractography of multi-shell diffusion-weighted imaging data using a multi-tissue model. *NeuroImage* **123,** 89–101 (2015).
60. Dhollander, T., Raffelt, D. & Connelly, A. Unsupervised 3-tissue response function estimation from single-shell or multi-shell diffusion MR data without a co-registered T1 image. in *Workshop on Breaking the Barriers of Diffusion MRI* (2016).
61. Garyfallidis, E. *et al.* Recognition of white matter bundles using local and global streamline-based registration and clustering. *NeuroImage* (2017). doi:10.1016/j.neuroimage.2017.07.015
