# Supplementary material for: The challenge of mapping the human connectome based on diffusion tractography
Source: Nat Commun. 2017 Nov 7;8:1349. doi: 10.1038/s41467-017-01285-x (PMC5677006; doi:10.1038/s41467-017-01285-x)
Supplement: Supplementary file 3 — Description of Additional Supplementary Files [file 41467_2017_1285_MOESM3_ESM.pdf]

File Name: Supplementary Software 1
Description: Source code of scoring system used

File Name: Supplementary Movie 1
Description: Examplary bottleneck situation in temporal lobe area of simulated phantom

File Name: Supplementary Movie 2
Description: Examplary bottleneck situation in temporal lobe area of in-vivo HCP data set

File Name: Supplementary Movie 3
Description: Visualization of the phantom raw diffusion-weighted images
